# Industrial point source emissions and incident breast and lung cancers: two case–control studies

**DOI:** 10.1007/s10552-026-02168-7

**Published:** 2026-04-15

**Authors:** Joseph Boyle, Patrick Nana-Sinkam, Kandace P. McGuire, Bernard F. Fuemmeler

**Affiliations:** 1 Department of Family Medicine and Population Health, Virginia Commonwealth University, 830 East Main Street, 6thFloor, Richmond, VA 23219 USA; 2 Massey Comprehensive Cancer Center, Richmond, VA USA; 3 Division of Pulmonary Disease and Critical Care Medicine, Virginia Commonwealth University, Richmond, VA USA; 4 Department of Surgery, Virginia Commonwealth University, Richmond, VA USA

**Keywords:** Environmental exposure, Air pollution, Air toxics, Epidemiology, Breast cancer, Lung cancer

## Abstract

**Purpose:**

Better understanding of the environment’s role in breast cancer (BC) and lung cancer (LC) incidence is crucial for population health. Growing evidence suggests environmental factors may be relevant for these cancers. We performed two case–control analyses to determine associations between industrial point source emissions, which are proxies for ambient industrial point source-based environmental exposures, and incident BC and LC.

**Methods:**

We obtained information for 5,801 female adults with BC, 5,250 adults with LC, and 47,956 cancer-free adult controls over 2010–2023. We linked individuals’ residences to releases of 11 agents reported to EPA’s Toxics Release Inventory, determining presence and number of facilities and inverse distance-weighted emissions using several geographic buffer distances and lag times. We fit conditional logistic regression models with multiple comparisons adjustments to datasets derived from three matching methods.

**Results:**

We identified positive associations between ethylene oxide and chromium exposures with BC. Associations were consistently positive for presence of ethylene oxide-emitting facilities within 5-20 km and chromium-emitting facilities from 5 to 20 km. We identified positive associations between formaldehyde and chromium exposures with LC. Associations were consistently positive for presence of formaldehyde-emitting facilities within 5–20 km and chromium-emitting facilities from 5 to 20 km.

**Conclusion:**

We observed relationships between industrial point source emissions with BC and LC, particularly for presence of facilities within 5 km of residence. Research should continue examining the role of such emissions for risk of these cancers.

**Supplementary Information:**

The online version contains supplementary material available at 10.1007/s10552-026-02168-7.

## Introduction

Lung cancer is the second most commonly diagnosed malignancy among adults in the US, and breast cancer diagnoses are the most common among women specifically. Among women, breast cancer rates have been slowly rising in the twenty-first century. Though some of the increase is attributable to diagnoses at localized stage, now one in eight women in the US will receive a breast cancer diagnosis in their lifetime [1, 2]. Racial and ethnic disparities for these cancers also exist. For example, Black men have a recent lung cancer incidence rate 10 percent higher than White men, and with incidence less coupled to smoking than White men [1, 3].

A small percentage (5–10%) of breast cancers develop through genetic inheritance [4], and genetic factors for lung cancer are relatively understudied [5]. Therefore, in addition to lifestyle behaviors, environmental exposures are important to consider for these cancers in order to identify risk factors contributing to unexplained incidence. Outdoor air pollution, and specifically particulate matter less than 2.5 microns in diameter (PM_2.5_), is classified as a known human carcinogen by the International Agency for Research on Cancer (IARC), drawing on robust evidence from mechanistic, epidemiological, and animal studies [6]. Numerous studies conducted in geographically diverse populations have identified associations between air pollution and cancer, and in particular, breast and lung cancer, that persist after adjusting for other risk factors [7–15]. Many of these studies have analyzed criteria air pollutants such as PM_2.5_ and nitrogen dioxide. Notably, the distribution of air pollution in the US is not uniform [9] and varies across demographics and socio-economic characteristics [16]. Despite this, the link between industrial point source emissions and risk of lung and breast cancers has not been well studied.

The development of databases tracking environmental exposures with high spatial precision, resolution, and temporal fidelity, coupled with access to large geocoded electronic health record (EHR) databases, permits the testing of a large set of exposures for evaluating associations with cancer risk. For example, a recent analysis of breast and colorectal cancer hotspots in Kentucky combined cancer incidence data from the state cancer registry with concentrations of several metallic air pollutants from the U.S. Environmental Protection Agency (EPA) to estimate concentrations at the residence for each individual diagnosed with cancer [17]. Additionally, analyses of breast cancer [18] and non-Hodgkin lymphoma [19] used environmental exposure scores from the Risk-Screening Environmental Indicators (RSEI) model developed by EPA and found them to be significant contributors to the risk of these cancers.

In this study, we combined geospatial proxies for ambient environmental exposure with EHR data to investigate the associations between these exposures and incident lung and breast cancers. Given previous studies linking emissions of various hazardous air pollutants to breast [17, 20–23] and lung [24] cancer risk, we hypothesized that proxies for ethylene oxide, nickel, and cadmium exposure would be associated with incident breast cancer, and that proxies for cobalt, nickel, and benzene exposure would be associated with incident lung cancer, with the strongest associations at the smallest geographic buffer distances.

## Methods

### Study design and outcome ascertainment.

We performed separate matched case–control studies of lung and breast cancers using data from a large cancer center in the southeast US. We obtained all instances of a first primary diagnosis for lung and female breast cancer at Massey Comprehensive Cancer Center (“Massey”) between January 1, 2010 and December 31, 2023. Additional inclusion criteria were that the patient resided in Virginia at the time of diagnosis, was at least 18 years old at the time of diagnosis, was not imprisoned, and had no previous cancer diagnoses. Regarding controls, we obtained a dataset of all primary care visits to Virginia Commonwealth University (VCU) Health system, the broader hospital system of which Massey is a part, during the same period (January 1, 2010 to December 31, 2023). Additional inclusion criteria for the controls were that the patient was at least 18 years old at the time of visit, was not imprisoned, and had no previous cancer diagnoses. If a control had multiple primary visits during this time frame, data from only the first visit was obtained. The control dataset also excluded any patient who later was diagnosed with lung or breast cancer at Massey during the time frame to remove the possibility of one person appearing in both datasets. There was no attrition, randomization, or power analysis conducted as this was a retrospective and observational study. Sex was considered a biological variable, used for restricting to only female breast cancer cases and matching on sex for the lung cancer analyses. See Supplemental Material for more information on the case and control datasets. This study was determined to be exempt by the Institutional Review Board at VCU.

### Covariates

For all patients, we recorded a set of covariates including age at visit (where visit meant the time of diagnosis for cases and primary care appointment for controls), sex, race, ethnicity, alcohol use, and tobacco use, which were defined by self-report. For the alcohol and tobacco variables, we categorized use into current, past, never, and unknown. We also obtained residential address at the visit date from the EHR and used ESRI’s ArcGIS Pro [25] to obtain geographic coordinates (latitude and longitude). Additionally, we linked geocoded addresses to census-tract level values of the Area Deprivation Index (ADI) [26, 27]. The ADI is a publicly available index that is constructed to estimate neighborhood socio-economic deprivation and contains 17 tract-level variables in the income, education, employment, and housing quality domains. We used a transformation of the ADI that is standardized to have mean 100 and standard deviation 20 [28] and used the ADI values for each participant based on the year of their visit date (see Supplemental Material).

### Exposures

The U.S. EPA produces and maintains the Toxics Release Inventory (TRI) [29, 30], a program under which industrial facilities report data annually on the types and quantities of chemical emissions if they meet certain regulatory requirements. These data have been reported to the TRI under the Emergency Planning and Community Right-to-Know Act since the 1980s. The EPA’s Risk-Screening Environmental Indicators (RSEI) [31] model applies chemical and exposure-route-specific toxicity weights and geographic information to TRI emissions data to produce the modeled hazard, which is a quantity that more specifically reflects emissions’ potential toxicity.

We obtained data from TRI/RSEI for 11 agents (metalloids, organic compounds, earth or transition metals, or inorganics) released and reported in the years 2005–2023 in or near Virginia, adding data from Maryland and North Carolina to allow for the possibility of emissions proximate to the Massey catchment area but not in Virginia. The vast majority of these data were releases to air; few transfers to water and off-site incineration were also included in this dataset. The agents for which we obtained data included: antimony and antimony compounds; arsenic and arsenic compounds; benzene; beryllium and beryllium compounds; cadmium and cadmium compounds; chromium and chromium compounds; cobalt and cobalt compounds; ethylene oxide, formaldehyde, hydrazine, and nickel and nickel compounds. We chose agents based on their listing as Group 1 (known) or 2A (probable) carcinogens according to IARC [6] and emissions during 2005–2023 in our study region. For simplicity, we refer to any agent named in the TRI/RSEI database “X and X compounds” in the TRI/RSEI database as “X” herein.

We geocoded the address of facilities reporting emissions using the facility identification information from EPA’s Facility Registry Service. Then, we calculated multiple exposure metrics using circular geographic buffers around participants’ residential address. For each agent, we calculated the presence of facilities, number of reporting facilities, and inverse-distance-weighted (IDW) RSEI modeled hazard of emissions using buffers of (2, 5, 10, 20) kilometers (km) based on recent literature [20, 24], and using reporting data from up to 5 years prior to the visit date. We considered the three emission types to assess the importance of existence, quantitative emission release, or both, of each agent, a variety of buffer sizes to allow varying importance of proximity based on fate and transport of different agents through the environment, and a variety of cumulative year lags to assess the varying temporal importance of each agent. This resulted in a maximum of 60 possible exposures (3 types × 4 buffer sizes × 5 cumulative year lags) for each agent. To decrease the impact of extremely low-prevalent exposures, we only considered those for which there were at least 10 exposed cases and controls. Finally, we did not consider continuous exposures to increase the interpretability of exposure contrasts and given the unclear meaning of, for example, combining multiple continuous distances for multiple facilities emitting the same agent.

### Statistical analysis

We compared distributions of variables between groups (breast and lung cancer cases and controls) using one-way analyses of variance for continuous variables and chi-squared tests for categorical variables. To maximize the comparability between the case and control samples, we used a variety of matching methods for each cancer analysis. We identified separate sets of controls for each cancer using three strategies: nearest-neighbor matching, exact matching, and optimal matching (see Supplemental Material for more information on the matching methods). We nearest-neighbor matched and exact-matched on age, sex, race, and year; and optimal-matched on age, sex, race, year, alcohol, tobacco, and ADI using the “MatchIt” [32] R package. We implemented the three matching methods with respect to their ability to control confounding and resultant sample size as well as differences in the scope of variables deemed necessary to reasonably match participants (see adjustment variables below). We compared associations by agent and compared effect sizes and p-values across analyses using each of the matching methods, requiring statistical significance across all matching methods to reflect variation in the view of important covariates and methods and lessen the possibility of false positives based on a specific choice of adjustment covariates or matching method.

Then, using each matched dataset, we fit a series of conditional logistic regression (CLR) models for each exposure. CLR models evaluate whether, conditional on the matching variables, differences in within-matched-set exposures are associated with odds of being the case. In the nearest-neighbor and exact-matched CLR models, we adjusted for alcohol and tobacco use and ADI. We also performed a sensitivity analysis after restricting the breast and lung cancer datasets to never-smokers and re-matching never-smoking controls to the cases. We performed another sensitivity analysis utilizing “negative control” exposures and found our results to be generally robust to these (see Supplemental Material).

### Inference and visualization

We defined overall statistical significance requiring a Bonferroni-adjusted (per tested agent) p-value of less than 0.01 across all three matching analyses. In other words, we corrected the p-values for multiple testing, and required a tested agent (combination of exposure type, buffer size, and cumulative year lag) to have less than a Bonferroni-corrected probability of 1% of truly being null across all three matching analyses to conclude overall significance. We produced volcano plots illustrating the relationship between effect size (logarithm of the odds ratio (OR) from CLR) and negative logarithm of the Bonferroni-adjusted p-value for each matched analysis and cancer type. We conducted statistical analyses in R, version 4.4.2 [33].

## Results

A total of 5,801 women were diagnosed with breast cancer during the study period; 5,250 individuals were diagnosed with lung cancer, and we sampled 47,956 cancer-free controls (Table 1). All individuals diagnosed with breast cancer and approximately 48% and 54% of the lung cancer and control distributions were female. Individuals identifying as White and Black were the most common (60% and 34% for breast; 63% and 33% for lung; 46% and 41% for controls, respectively). Most individuals did not identify as Hispanic. Individuals with lung cancer were older at diagnosis (median = 66 years, inter-quartile range (59, 74) years) than women with breast cancer (60, 51, 68) and controls (42, 29, 54). Among women with breast cancer, 75% were estrogen receptor (ER)-positive, 64% were progesterone receptor (PR)-positive, and 12% were HER-2 positive. Body Mass Index (BMI) was lowest among those with lung cancer (median = 25.6 kg/m^2), compared to those with breast cancer (28.8) and controls (28.2). Controls were most likely to report alcohol use at visit (36%), and individuals with lung cancer were the most likely to report tobacco use at visit (23%). Matching provided adequate balance to the case and control distributions (see Supplemental Material for details on matching results and Table S1 for demographic, clinical, and lifestyle characteristics by cancer and matching analysis). Additional information regarding the geographic distributions of facilities emitting tested agents and the study sample is provided in Figure S29.

**Table 1:** Comparison of demographic, clinical, and lifestyle characteristics of adult individuals diagnosed with breast cancer, lung cancer, or cancer-free controls by cancer type

*Variable*	Breast Cancer (N = 5,801)	Lung Cancer (N = 5,250)	Control (N = 47,956)	P–value
*Sex*				
Female	5801 (100)	2511 (48)	26,057 (54)	< 0.001
Male	0 (0)	2739 (52)	21,856 (46)	
Other/Unknown	0 (0)	0 (0)	43 (0)	
*Race*				
AI/AN	12 (0)	6 (0)	115 (0)	< 0.001
Asian	92 (2)	44 (1)	1258 (3)	
Black	1989 (34)	1748 (33)	19,704 (41)	
Multiple	32 (1)	14 (0)	340 (1)	
Native Hawaiian/PI	0 (0)	0 (0)	13 (0)	
Unknown/Other	180 (3)	153 (3)	4324 (9)	
White	3496 (60)	3285 (63)	22,202 (46)	
*Ethnicity*				
Hispanic	88 (2)	48 (1)	1735 (4)	< 0.001
Not Hispanic	5675 (98)	5179 (99)	45,229 (94)	
Unknown	38 (1)	23 (0)	992 (2)	
*Age*	60.0 [51.0, 68.0]	66.0 [59.0, 74.0]	42.0 [29.0, 54.0]	< 0.001
*Primary Payer*				
Medicaid	421 (7)	527 (10)	5487 (11)	< 0.001
Medicare	2038 (35)	2811 (54)	5211 (11)	
Private	2527 (44)	986 (19)	21,238 (44)	
Uninsured	376 (6)	508 (10)	0 (0)	
Unknown	362 (6)	349 (7)	15,567 (32)	
VA/TRICARE	77 (1)	69 (1)	453 (1)	
*ER–Status*				
Positive	4361 (75)	–	–	–
Negative	1272 (22)			
Borderline/Unknown	168 (3)			
*PR–Status*				
Positive	3725 (64)	–	–	–
Negative	1856 (32)			
Unknown	220 (4)			
*HER–2 Status*				
Positive	718 (12)	–	–	–
Negative	4006 (69)			
Borderline/Unknown	1077 (19)			
*Grade*				
1	536 (9)	181 (3)	–	< 0.001
2	1217 (21)	361 (7)		
3	1089 (19)	613 (12)		
4	6 (0)	33 (1)		
Unknown	2953 (51)	4062 (77)		
*Body Mass Index*	28.8 [24.5, 33.9]	25.6 [22.1, 29.9]	28.2 [24.1, 33.8]	< 0.001
*Alcohol Use*				
Current	1387 (24)	1333 (25)	17,251 (36)	< 0.001
Past	292 (5)	762 (15)	3106 (6)	
Never	1064 (18)	1137 (22)	11,778 (25)	
Unknown	3058 (53)	2018 (38)	15,821 (33)	
*Tobacco Use*				
Current	386 (7)	1199 (23)	8466 (18)	< 0.001
Past	945 (16)	2227 (42)	6566 (14)	
Never	1979 (34)	454 (9)	20,630 (43)	
Unknown	2491 (43)	1370 (26)	12,294 (26)	
Area Deprivation Index	100.5 [88.3, 112.7]	106.0 [93.8, 118.1]	104.3 [93.4, 119.1]	< 0.001

AI/AN, American Indian/Alaska Native; PI, Pacific Islander; VA, Veterans Affairs; ER = Estrogen Receptor; PR, Progesterone Receptor; HER-2 denotes a protein that helps breast cancer cells grow quickly; Categorical variables presented with count and frequency. Continuous variables presented with median [inter-quartile range] among non-missing values. Percentages may not sum to 100 due to rounding. Area Deprivation Index is standardized to have mean 100. P-values tested with one-way analysis of variance for continuous variables and chi-squared test for categorical variables (tests excluded Other/Unknown category if applicable and tests for grade excluded controls)

### Breast cancer analysis

**Fig. 1:**
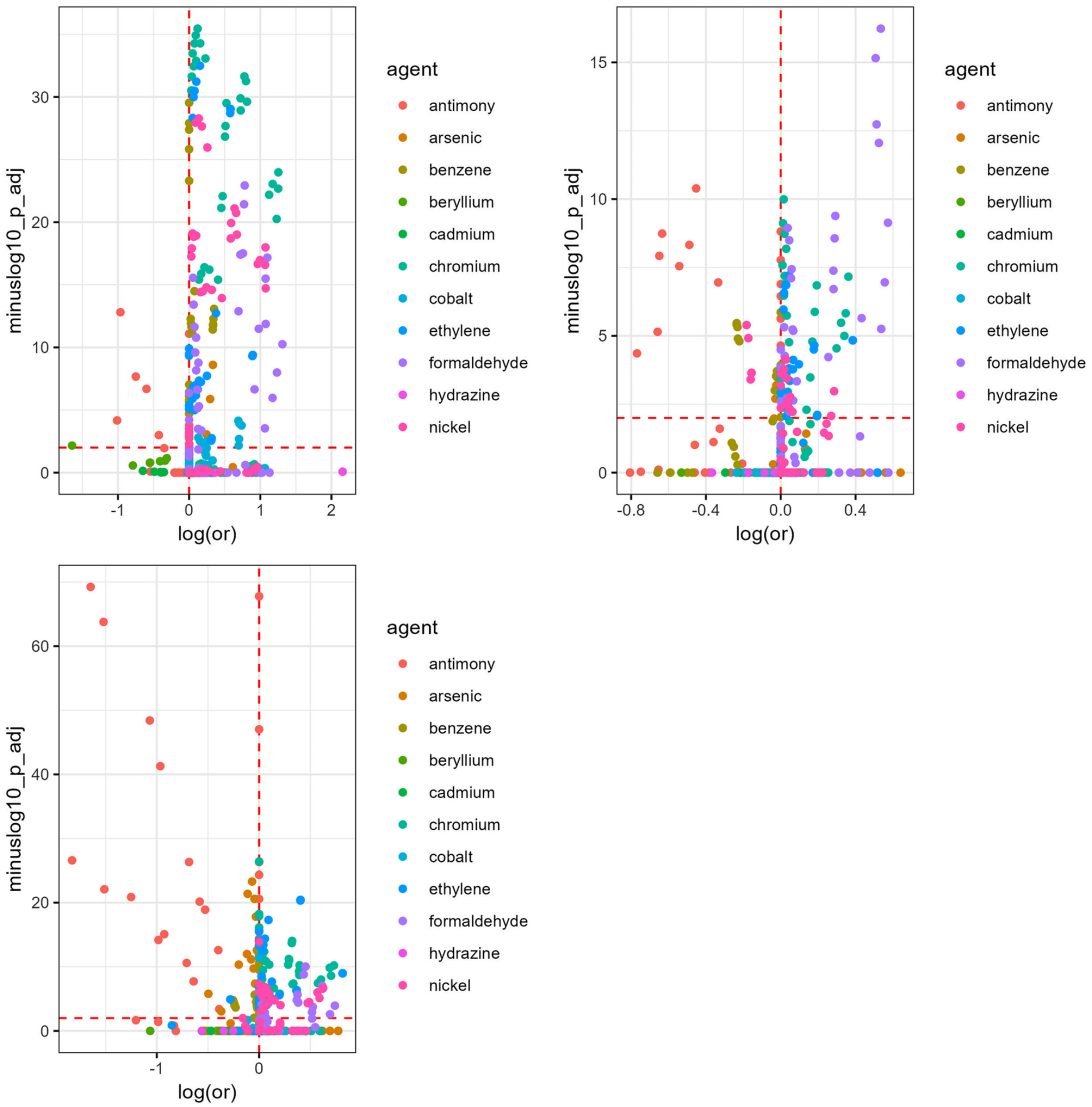
Volcano plot for point source industrial emissions and breast cancer, by matching analysis. Plot panels are from nearest neighbor, exact (top row), and optimal matching (bottom row) from left to right. In the volcano plot, the x-axis is the logarithm of the odds ratio estimated by the conditional logistic regression model, and the y-axis is the negative logarithm of the Bonferroni-adjusted p-value for the coefficient. The red horizontal dashed line represents the negative logarithm of the significance threshold (0.01), and the red vertical line represents a logarithm of the odds ratio equal to zero. As such, points above the horizontal dashed line denote exposures representing a matching analysis’ tested exposure identified as significant based on the multiple comparisons-adjusted p-value

Breast cancer cases tended to have more ethylene oxide exposures than controls, particularly at 20 km thresholds (Figure S8). The number of benzene exposures at 10 km and 20 km (Figure S3) and number of cobalt exposures at 20 km (Figure S7) were slightly higher among controls. The nearest neighbor matching method yielded elevated and statistically significant associations for at least half of the exposures tested for ethylene oxide, chromium, and formaldehyde (Table 2). The same was true for formaldehyde in the exact matching method, and ethylene oxide and chromium for the optimal matching method. The nearest neighbor method tended to estimate the largest effect sizes and smallest p-values of the three matching methods (Fig. 1, Figure S23).

**Table 2:** Proportion of statistically significant and positive associations between point source industrial emissions with incident breast and lung cancers, by matching approach

Agent	Breast cancer			Lung cancer		
	Nearest-Neighbor	Exact	Optimal	Nearest-Neighbor	Exact	Optimal
Ethylene Oxide	63	53	67	47	40	40
Formaldehyde	50	50	30	72	75	50
Benzene	38	12	8	27	18	22
Chromium	50	38	50	75	67	50
Nickel	48	23	30	55	32	40
Arsenic	10	0	0	0	3	0
Cobalt	18	0	0	56	36	18

Numbers in the table are given as percentages. Significance is based on Bonferroni-adjusted p-values. Hydrazine, antimony, beryllium, and cadmium are omitted from the table due to an absence of any significant Bonferroni-adjusted p-values for any method or cancer

Across all matching methods, ethylene oxide and chromium presented the most consistent evidence of positive associations (Table 2, Figure S25), with overall statistically significant and elevated findings across distance thresholds from 5 to 20 km (Table S2). Most ethylene oxide associations were with exposures estimated within 5 km (43% of significant ethylene oxide associations) and 10 km (70%) of the residence. Of the elevated chromium associations, most were within 5 km (35% of significant chromium associations) and 10 km (57%). Additionally, formaldehyde had several elevated associations relating to presence and number of facilities. Among these, the largest associated mean ORs were for the presence of formaldehyde facilities (range of ORs = 2.29–2.40) and chromium facilities (range of ORs = 1.95–2.17) within 5 km, as well as the presence of formaldehyde facilities within 10 km (range of ORs = 1.96–2.10) and presence of ethylene oxide facilities within 5 km (range of ORs = 2.00–2.01) (Figure S27). Typically, associations attenuated as buffer sizes increased. There were also elevated associations for the ethylene oxide IDW emissions within 5 km. There were few overall statistically significant and inverse associations, most of which were for antimony at the largest buffer (20 km).

Restricting the breast cancer analysis to never-smokers (34% of breast cancer cases) yielded similar associations with chromium, with a positive relationship observed across all distance thresholds (Table S3, Table S4) and all but one agent-specific statistically significant association from the primary analysis maintained, with similar estimated magnitudes of association but less consistent evidence of significant associations with ethylene oxide and formaldehyde due to the decreased sample size.

### Lung cancer analysis

**Fig. 2:**
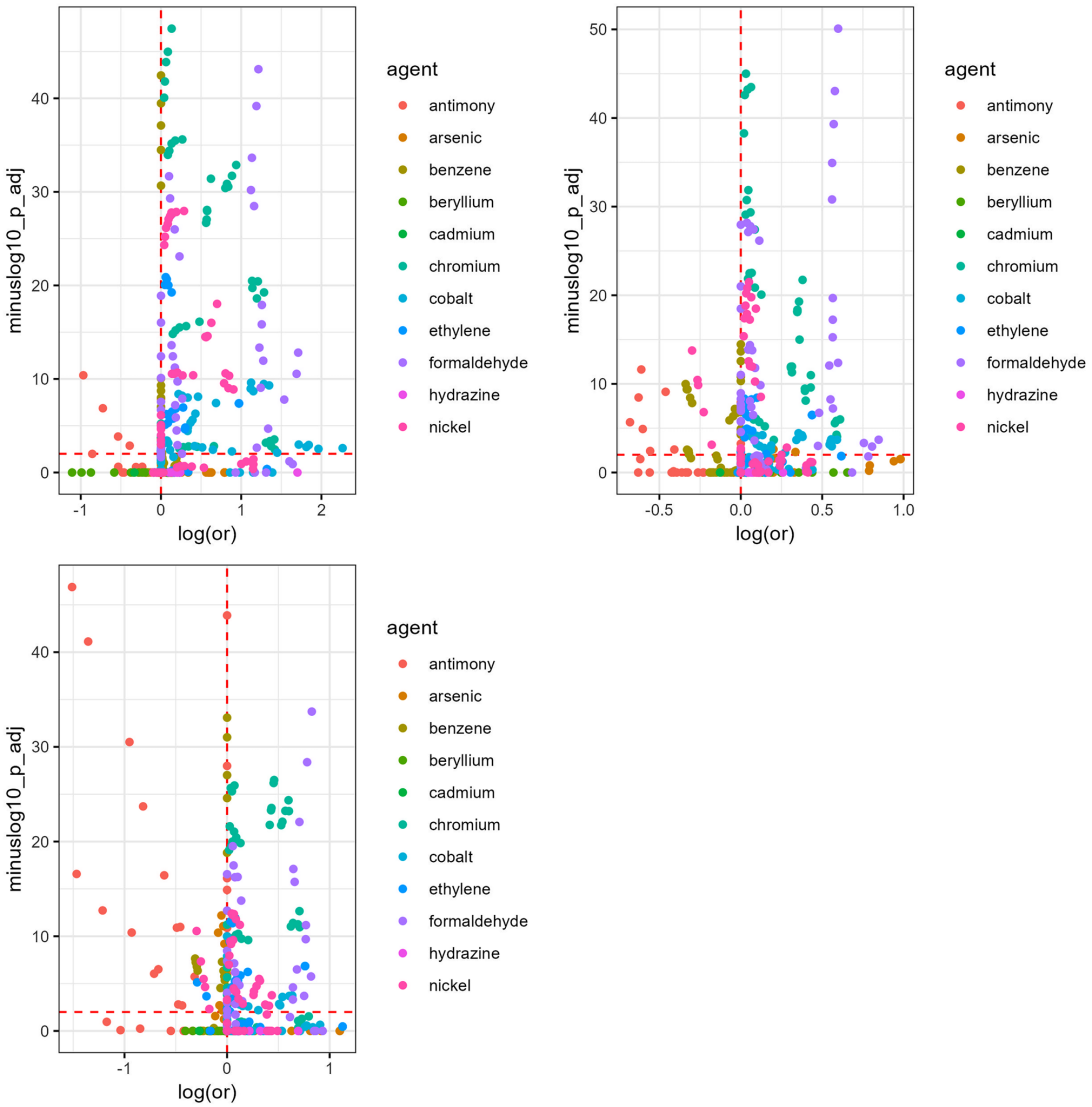
Volcano plot for point source industrial emissions and lung cancer, by matching analysis. Plot panels are from nearest neighbor, exact (top row), and optimal matching (bottom row) from left to right. In the volcano plot, the x-axis is the logarithm of the odds ratio estimated by the conditional logistic regression model, and the y-axis is the negative logarithm of the Bonferroni-adjusted p-value for the coefficient. The red horizontal dashed line represents the negative logarithm of the significance threshold (0.01), and the red vertical line represents a logarithm of the odds ratio equal to zero. As such, points above the horizontal dashed line denote exposures representing a matching analysis’ tested exposure identified as significant based on the multiple comparisons-adjusted p-value

Visualizing the exposure distributions (Figures S12-S22), the number of benzene (Figure S14) and cobalt (Figure S18) exposures were slightly higher among controls. The nearest neighbor matching method found elevated and statistically significant associations for at least half of the exposures tested for chromium, formaldehyde, cobalt, and nickel (Table 2). The same was true for chromium and formaldehyde in the exact and optimal matching methods. The nearest neighbor method tended to estimate the largest effect sizes of the three matching methods (Fig. 2, Figure S24).

Across all three matching methods, chromium and formaldehyde presented the most consistent evidence (Table 2, Figure S26), with overall statistically significant and elevated findings across all distance thresholds from 5 to 20 km (Table S2). Of the statistically significant chromium findings, many associations were within 5 km (33%) and 10 km (67%) of residence. Of the formaldehyde findings, most were from buffers of within 10 km (50%). Among the positive associations, the largest mean ORs were for the presence of formaldehyde facilities within 5 km (range of ORs = 2.71–2.83) and 10 km (range of ORs = 2.20–2.37), as well as the presence of chromium facilities within 5 km (range of ORs = 2.05–2.24) (Figure S28). Typically, associations attenuated as buffer sizes increased. Elevated associations were identified using the IDW modeled hazard, including benzene within 5 km, 10 km, and 20 km, and ethylene oxide within 10 km. There were few overall statistically significant and inverse associations, all of which were from antimony at the largest buffer (20 km).

Restricting the analysis to never-smokers (9% of lung cancer cases) resulted in fewer statistically significant and elevated findings, but with similar estimated magnitudes of association and with some remaining from chromium and formaldehyde facility presence and number within 20 km (Table S3, Table S4).

## Discussion

We performed two case–control studies utilizing a large EHR database from a comprehensive cancer center to investigate whether ambient environmental exposures were associated with incident breast and lung cancer. Broadly, we observed positive associations between ethylene oxide and chromium emissions with breast cancer, and formaldehyde and chromium emissions with lung cancer.

Our findings contribute to the literature surrounding environmental exposures and breast cancer risk. For example, exposure to ethylene oxide in occupational contexts [22] and ambient environmental contexts within 10 km [21] has been associated with increased breast cancer mortality and risk, respectively. In our study, we found that the presence and number of ethylene oxide-emitting facilities within 5 km and 10 km were associated with elevated breast cancer risk, with the largest odds ratios of these coming from the 5 km presence metric. Our sample had a similar overall proportion of participants living within 5 km of an ethylene oxide-emitting facility (5.5%) to the above-mentioned study (5.7%) in the NIH-AARP Diet and Health Study cohort. Similar to that study, which included different geographic areas than in our analysis, we also observed disparities in exposure to ethylene oxide-emitting facilities by race, with Black women more likely to reside within 5 km of such a facility (9.1%) than White women (3.2%). Ethylene oxide is most commonly emitted by commercial sterilization facilities, chemical plants, and medical facilities [34]. It degrades relatively slowly upon release to air, with an estimated half-life of several months [35–37] and transport demonstrated across several kilometers from the emission location [38, 39], which is consistent with our observations of associations out to 20 km. It is genotoxic as an alkylating agent [40, 41] and there is mixed evidence as to whether it has endocrine-disrupting properties [42]. Our findings contribute to the limited prior evidence of associations between airborne ethylene oxide emissions and breast cancer risk, although the mechanism of action remains unclear.

Additionally, we found that presence and number of chromium-emitting facilities at 5 km and number at 10 km were associated with higher breast cancer risk. Though relatively understudied, the relation of breast cancer risk with ambient exposure to metals including chromium through air has been suggested in some prior studies. Higher chromium levels have been found in the toenail clippings of women with postmenopausal breast cancer compared to controls [43], and chromium contributed to a principal-component mixture of metals that was significantly associated with breast cancer risk [44]. Additionally, a recent analysis of breast cancer cases in Kentucky found that the odds of residing in a breast cancer spatial hotspot were elevated with increasing concentrations of chromium, arsenic, cadmium, and nickel [17]. Further, there is evidence supporting the relationship between residential proximity and embodied exposure. A recent analysis of metal concentrations in the toenails of women in the Sister Study cohort identified that proximity to chromium-emitting facilities was associated with toenail chromium levels among Black women [45]. Separate analyses of data from the same study have found that an overall weighted index of exposure was associated with postmenopausal breast cancer risk [23], and that emissions of nickel compounds and trichloroethylene within 3 km of the residence were significantly associated with breast cancer risk [46]. Regarding mechanisms, certain metal or metalloid air pollutants including chromium, cadmium, and arsenic have been demonstrated to damage DNA [47–49] or bind to estrogen receptors, which could promote the development of breast tumors [50–52]. Notably, many of the chromium findings remained in the sensitivity analysis restricting breast cancer cases to never-smokers; comparatively fewer ethylene oxide findings remained, though with similar estimated magnitudes of association. These findings may suggest chromium to relate to risk independent of smoking status and ethylene oxide to interact with smoking status in modifying risk. However, our study was not designed to address such differential roles and future research should investigate the interaction of air pollution exposure, smoking status, and breast cancer risk.

We also identified several notable findings in the lung cancer analysis. Although the influence of environmental exposures, including those that we studied, was not as prominent as that of tobacco smoking on lung cancer risk [53], the contribution of environmental exposures to lung cancer incidence among non-smokers remains unclear [54, 55], thus motivating additional investigation on the role of industrial point source air emissions. A recent analysis in the NIH-AARP cohort reported elevated risk for lung cancer with cobalt and beryllium exposures from the TRI within 5 km and 10 km [24]. Additionally, a large ecological study conducted using TRI data identified positive associations of chromium, formaldehyde, and nickel emissions with lung cancer in non-metropolitan counties [56]. We found that the presence and number (chromium) and presence, number and IDW emissions (formaldehyde) were significantly associated with lung cancer risk. Among these, we identified the strongest signal from the presence of facilities emitting these agents within 5 km of the residence. While the highest exposures to chromium often occur through occupational settings such as chrome plating or chromate production [57], we found some relevance to the general population living in close proximity through ambient releases. There have been relatively few cohort studies of lung cancer risk based on formaldehyde exposures in occupational settings, with one [58] identifying increased risk and two [59, 60] identifying no increased risk. Our data suggest that continued investigation of these exposures is important, given that the proportion of lung cancers diagnosed in never-smokers has increased over time [61]. The sensitivity analysis restricting lung cancer cases to never-smokers revealed certain chromium and formaldehyde associations despite a considerable reduction in the sample size, and future research should investigate the role of air pollution exposures independently of and interacting with smoking status in lung cancer risk.

Our study has numerous strengths. We utilized a large EHR from a major comprehensive cancer center over a long time period that captured not only the demographic characteristics but also the geographic distribution of individuals diagnosed with breast cancer, lung cancer, and cancer-free controls. The large sample size afforded by this dataset increased statistical power to detect associations with ambient environmental exposures. Additionally, we employed a unique exposure assessment approach that was informed by geospatial and data science techniques that considered a broad range of environmental exposures – varying the granularity of emissions included from presence of facilities to number of facilities to inverse distance- and toxicity-weighted emissions – as well as a varying set of geographic buffers between residential addresses and emitting facility addresses. This process drew upon the rich data sources of the TRI database and RSEI model and avoided a priori restriction to specific geographic buffers and exposure types for each tested agent owing to a relative paucity of data on ambient exposures to these agents through industrial emissions. A stated goal of the RSEI model is to “help establish priorities for further investigation” [62] and is thus a natural complement to EHR databases to investigate the role of environmental exposure proxies for cancer incidence. While the RSEI model is not designed explicitly for use in etiologic analyses, the consistency in our findings across multiple exposure metrics and matching strategies support the RSEI model’s design intent as a “starting point to identify situations of potential concern that may warrant further investigation” [63]. This consistency required drawing a conclusion of statistical significance across a set of three distinct matching methods (one approach emphasizing within-neighborhood socio-economic status comparisons, and others allowing broader such contrasts, all of which matched on essential factors including sex and age) in order to limit the likelihood of spurious findings that were unique to a single matching method. Collectively, this process in the future will ultimately inform downstream analyses that are more targeted with regard to the particular exposures specified, in order to design effective interventions including policy related to ambient environmental exposures that decrease the modifiable burden of these cancers. Finally, we utilized an extensive set of individual- and neighborhood-level covariates, including demographics, alcohol and tobacco use, and neighborhood disadvantage as measured by the ADI, through either matching or adjustment in order to minimize the possibility of confounding.

The strengths of our study should be viewed in the context of its limitations. First, we had access to only the residential address of study participants at the time of their visit. It is possible that patients in our study may have lived at a different address over the time period considered in our study (up to the five years prior to the visit), or have moved closer to care and/or with relatives by the time of diagnosis, in which case a degree of exposure misclassification would result. However, residential mobility does not appear to be a major factor for older adults. A recent analysis of American Community Survey data estimated that slightly less than 10 percent of adults aged 60–69 in the US had moved in the past year, implying that address at visit date may be a reasonable assumption for recent exposures [64]. Second, our exposure assignment was based on proximity to emitting facilities, in the absence of serviceable direct measures such as biomarkers. However, proximity is a standard exposure proxy [20, 24] in large and/or hypothesis-generating studies such as ours. Also, there is some evidence to suggest that proximity is a reasonable proxy for true exposure for some chemicals, especially at close proximity. For example, the levels of hemoglobin adducts in non-smokers who lived within a half-mile of an ethylene oxide-emitting facility in Illinois were greater than those measured in a comparison population of non-smokers who lived farther from the facility [65]. These so-called “meet-in-the-middle methods” that relate biomarker levels with contributors to the external exposome can identify relevant biological pathways to better understand the underlying mechanisms [66–68]. Future research should verify that closer proximity drives a greater degree of embodied exposure for these and similar findings, for example, through blood or nail samples as described above. It will also be necessary to determine reliable biomarkers (beyond, say, DNA adducts) of long-term exposure to specific air pollutants of interest [69]. Third, we did not have access to data on every major risk factor for the cancers under study. For example, we lacked certain information (particularly for controls) on variables that may be important for incidence of these cancers, including breastfeeding status, parity, and age at menarche [70], the *BRCA* mutation [71], or hormone replacement therapy [72], for breast cancer and family history [73] or chronic respiratory disease [74] for lung cancer. Also, screening information was not available; however, the relatively long time period of our study makes it likely that a patient who is cancer-free at the primary care visit but develops cancer within the time period would ultimately be included in the case and not control dataset. Relatedly, missingness in the BMI variable prevented us from being able to match on or adjust for this factor, but there were similar distributions of the observed BMI values in the matched datasets. Fourth, it is possible that the widest of our geographic buffers (20 km) may have extended beyond the true maximum range of emissions from facilities leading to embodied exposure, in which case true exposures would not occur. However, 20 km is smaller than the maximum radius considered in RSEI’s calculation of modeled toxicity concentrations (50 km), and we noted that the majority of our statistically significant findings and the largest associated ORs occurred at narrower (e.g., 5 km, 10 km) buffers. Fifth, because our study is observational and cross-sectional in nature, we were unable to draw conclusions regarding causality, and we cannot rule out the possibility of residual confounding by unmeasured covariates. Sixth, the conclusions of our study are based on the frequency of significant tested exposures, in contrast to a pooled overall estimate akin to that provided by a meta-analysis. In future targeted analyses we intend to produce highly interpretable effect estimates for identified environmental exposures relevant in the etiology of lung and breast cancer.

In conclusion, we identified evidence for the role of ethylene oxide and chromium emissions for the development of breast cancer and formaldehyde and chromium emissions for lung cancer. These findings were generally robust to type and geographic proximity to the emissions and choice of comparison population of cancer-free controls. Future research should seek to replicate these findings, directly measure environmental exposures using biomarkers, examine these relationships by cancer subtype and aggressiveness, and design and maintain prospective and diverse cohorts to elucidate the specific timing and mechanistic pathways through which these exposures may promote carcinogenesis.

## Supplementary Information

Below is the link to the electronic supplementary material.

Supplementary file1 (DOCX 4505 KB)

## Data Availability

The data analyzed in this study are remotely stored in a secure platform at the corresponding author’s institution. Clinical data cannot be shared due for confidentiality and ethical reasons. Environmental exposures data were sourced from the EPA’s Toxics Release Inventory and Risk-Screening Environmental Indicators model, which are freely available online.
