# Supplementary material for: Industrial point source emissions and incident breast and lung cancers: two case–control studies
Source: Cancer Causes Control. 2026 Apr 15;37(5):81. doi: 10.1007/s10552-026-02168-7 (PMC13079530; doi:10.1007/s10552-026-02168-7)
Supplement: Supplementary file 1 — Supplementary file1 (DOCX 4505 KB) [file 10552_2026_2168_MOESM1_ESM.docx]

**Supplemental Material**

*“Industrial point source emissions and incident breast and lung cancers: two case-control studies”*

**Supporting Text – Methods**

Primary care visits were determined using scheduling department data that mentioned “family” or “primary medicine”. Visits to the VCU Health clinics at two other locations in different geographic areas in Virginia and Covid-specific clinic visits were excluded.

We use three sets of ACS 5-year estimates underlying the construction of the ADI based on the time period of our study. Specifically, we accessed ADI scores for the year 2019-2023, 2014-2018, and 2009-2013. For case/control participants with year of diagnosis/visit between 2019 and 2023, we assigned tract-level ADI values based on the 2019-2023 ACS 5-year estimates, which increase stability in the estimates in contrast to, for example, 1-year estimates. We performed a similar process for participants with year of diagnosis/visit between 2014 and 2018 (the underlying 2014-2018 ACS 5-year estimates), and between 2010-2013 (the underlying 2009-2013 ACS 5-year estimates). The ADI values are constructed in reference to all geographic areas in Virginia, due to the Virginia-based nature of our sample.

In our context, nearest-neighbor matching pairs the “closest” control patient to a case patient, where closeness is calculated by propensity scores. Nearest-neighbor matching is “greedy” in that it chooses the first closest match for a given unit. This method resulted in a 1/1 case/control ratio for the analytic sample. Exact matching creates unique strata wherein all controls matching a given case exactly on the matching variables are identified. This method creates identical covariate distributions with respect to the matching variables and results in a varying ratio of cases to controls per stratum. Optimal matching has a similar goal to nearest-neighbor but differs by minimizing a summary criterion – the sum of distances over all matching variables in the sample [1], [2].

To evaluate the quality of each matching method, we examined the standardized mean difference (SMD) for the matching variables and overall propensity distance as well as the number of matched units for each method. We considered the matching to adequately standardize the covariate distributions if the SMDs for the matching variables in the matched sample were less than 0.1. In the sensitivity analyses, we removed the tobacco use variable from the adjustment set for the nearest-neighbor and exact-matched CLR models.

We performed a sensitivity analysis utilizing “negative control” exposures [3] with pseudo-locations of pseudo-industrial facilities, with the aim of demonstrating differences in the intensity of the signal of our primary analysis with these pseudo-locations. For each cancer analysis, we generated the same number of exposures that were tested in the primary analysis. In each iteration, we randomly sampled the geographic location of a participant in the matched datasets to constitute the pseudo-location (e.g., binary presence/absence metric at the pseudo-location). For these, we considered in the sampling only participants with residential locations greater than 15km from emitting facilities in the primary analysis to avoid overlap with the true studied exposures. For each iteration, we randomly sampled one of (2km, 5km, 10km, and 20km) geographic buffers, created the buffer around the location, and assigned the negative control exposure to the dataset. We fit all three matching analyses and determined overall significance in the same way as in the primary analysis: a Bonferroni-corrected p-value of less than 0.1 and positive odds ratio across all three matching analyses.

**Supporting Text – Results**

Prior to matching, the breast cancer and control datasets were imbalanced, with an overall SMD of 1.35 for the propensity score distance. The nearest neighbor method provided adequate matching, with a matched overall SMD of 0.03 and all breast cancer cases matched to a control. Similarly, the exact method provided adequate matching, with a matched overall SMD of 0 (by definition); this method matched approximately 92% of the cases. The optimal matching method resulted in an overall matched SMD of 0.18, however only one variable (year, 0.12) had an individual SMD of greater than 0.10 in the matched sample, and all breast cancer cases matched to a control.

Prior to matching, the lung cancer and control datasets were imbalanced, with an overall SMD of 1.20 for the propensity score distance. The nearest neighbor method provided adequate matching, with a matched overall SMD of 0.02 and all lung cancer cases matched to a control. Similarly, the exact method provided adequate matching, with a matched overall SMD of 0 (by definition); this method matched approximately 87% of the cases. The optimal matching method resulted in an overall matched SMD of 0.22, however only the year and age variables had an individual SMD of greater than 0.10 (0.12 for both) in the matched sample, and all lung cancer cases matched to a control.

We found that the negative control analysis demonstrated robustness of our results to the pseudo-locations. Specifically, for the breast cancer analysis, only 16 pseudo-locations were identified as overall significant, which is five times fewer than the number identified in the primary analysis (Table S2). The identified overall significant pseudo-locations generally clustered in two counties outside the Richmond metropolitan area (Hanover and New Kent), and not proximate to any facility emitting ethylene oxide. For the lung cancer analysis, only 20 pseudo-locations were identified as overall significant, which is six times fewer than the number identified in the primary analysis (Table S2). The identified overall significant pseudo-locations generally clustered in the same two counties as above, and not proximate to any facility emitting formaldehyde. Therefore, the large difference in signal between the tested exposures in the primary analysis and the pseudo-locations in the sensitivity analysis support the robustness of the findings.

**References**

[1] B. B. Hansen and S. O. Klopfer, “Optimal full matching and related designs via network flows,” *J. Comput. Graph. Stat.*, vol. 15, no. 3, pp. 609–627, 2006.

[2] X. S. Gu and P. R. Rosenbaum, “Comparison of multivariate matching methods: Structures, distances, and algorithms,” *J. Comput. Graph. Stat.*, vol. 2, no. 4, pp. 405–420, 1993.

[3] M. Lipsitch, E. T. Tchetgen, and T. Cohen, “Negative control exposures in epidemiologic studies,” *Epidemiology*, vol. 23, no. 2, pp. 351–352, 2012.

**Table S1.** Comparison of demographic, clinical, and lifestyle characteristics of adult individuals diagnosed with breast cancer, lung cancer, or cancer-free controls by cancer type and matching analysis.

| *Variable* | **Breast Cancer/Nearest Neighbor** | | **Breast Cancer/Exact** | | **Breast Cancer/Optimal** | |
| --- | --- | --- | --- | --- | --- | --- |
|  | **Case (N=5801)** | **Control (N=5801)** | **Case (N=5354)** | **Control (N=15186)** | **Case (N=5801)** | **Control (N=5801)** |
| *Sex* | | | |  |  |  |
| Female | 5801 (100) | 5801 (100) | 5354 (100) | 15186 (100) | 5801 (100) | 5801 (100) |
| *Race* | | | |  |  |  |
| AI/AN | 12 (0.2) | 0 (0.0) | 0 (0.0) | 0 (0) | 12 (0.2) | 0 (0.0) |
| Asian | 92 (1.6) | 90 (1.6) | 40 (0.7) | 52 (0.3) | 92 (1.6) | 112 (1.9) |
| Black | 1989 (34.3) | 1991 (34.3) | 1802 (33.7) | 7486 (49.3) | 1989 (34.3) | 2072 (35.7) |
| Multiple | 32 (0.6) | 21 (0.4) | 2 (0.0) | 2 (0.0) | 32 (0.6) | 35 (0.6) |
| Unknown/Other | 180 (3.1) | 189 (3.3) | 144 (2.7) | 349 (2.3) | 180 (3.1) | 224 (3.9) |
| White | 3496 (60.3) | 3510 (60.5) | 3366 (62.9) | 7297 (48.1) | 3496 (60.3) | 3358 (57.9) |
| *Age* | 60.0 [51.0, 68.0] | 59.0 [50.0, 68.0] | 59.0 [50.0, 67.0] | 50.0 [42.0, 59.0] | 60.0 [51.0, 68.0] | 60.0 [50.0, 69.0] |
| *Body Mass Index* | 28.8 [24.5, 33.9] | 29.3 [24.7, 35.1] | 28.9 [24.5, 34.0] | 30.4 [25.1, 36.5] | 28.8 [24.5, 33.9] | 28.9 [24.3, 34.6] |
| *Alcohol Use* | | | |  |  |  |
| Current | 1387 (23.9) | 1968 (33.9) | 1306 (24.4) | 4753 (31.3) | 1387 (23.9) | 1529 (26.4) |
| Past | 292 (5.0) | 420 (7.2) | 262 (4.9) | 926 (6.1) | 292 (5.0) | 300 (5.2) |
| Never | 1064 (18.4) | 1725 (29.7) | 936 (17.5) | 3677 (24.2) | 1064 (18.4) | 1218 (21.0) |
| Unknown | 3058 (52.7) | 1688 (29.1) | 2850 (53.2) | 5830 (38.4) | 3058 (52.7) | 2754 (47.5) |
| *Tobacco Use* | | | |  |  |  |
| Current | 386 (6.6) | 905 (15.6) | 368 (6.9) | 2532 (16.7) | 386 (6.6) | 417 (7.2) |
| Past | 945 (16.3) | 1103 (19.0) | 874 (16.3) | 2205 (14.5) | 945 (16.3) | 1002 (17.3) |
| Never | 1979 (34.1) | 2647 (45.6) | 1799 (33.6) | 5894 (38.8) | 1979 (34.1) | 2174 (37.5) |
| Unknown | 2491 (42.9) | 1146 (19.8) | 2313 (43.2) | 4555 (30.0) | 2491 (42.9) | 2208 (38.1) |
| *ADI* | 100.5 [88.3, 112.7] | 101.6 [92.2, 113.8] | 100.5 [88.3, 112.6] | 106.6 [95.7, 120.2] | 100.5 [88.3, 112.7] | 100.0 [89.8, 111.9] |
| *Variable* | **Lung Cancer/Nearest Neighbor** | | **Lung Cancer/Exact** | | **Lung Cancer/Optimal** | |
|  | **Case (N=5250)** | **Control (N=5250)** | **Case (N=4543)** | **Control (N=15957)** | **Case (N=5250)** | **Control (N=5250)** |
| *Sex* | | | | | | |
| Female | 2511 (47.8) | 2652 (50.5) | 2207 (48.6) | 8191 (51.3) | 2511 (47.8) | 2527 (48.1) |
| Male | 2739 (52.2) | 2598 (49.5) | 2336 (51.4) | 7766 (48.7) | 2739 (52.2) | 2723 (51.9) |
| *Race* | | | | | | |
| AI/AN | 6 (0.1) | 8 (0.2) | 0 (0.0) | 0 (0.0) | 6 (0.1) | 7 (0.1) |
| Asian | 44 (0.8) | 48 (0.9) | 9 (0.2) | 10 (0.1) | 44 (0.8) | 53 (1.0) |
| Black | 1748 (33.3) | 1696 (32.3) | 1470 (32.4) | 6495 (40.7) | 1748 (33.3) | 1853 (35.3) |
| Multiple | 14 (0.3) | 7 (0.1) | 0 (0.0) | 0 (0.0) | 14 (0.3) | 16 (0.3) |
| Unknown/Other | 153 (2.9) | 162 (3.1) | 83 (1.8) | 165 (1.0) | 153 (2.9) | 157 (3.0) |
| White | 3285 (62.6) | 3329 (63.4) | 2981 (65.6) | 9287 (58.2) | 3285 (62.6) | 3164 (60.3) |
| *Age* | 66.0 [59.0, 74.0] | 66.0 [59.0, 74.0] | 65.0 [58.0, 72.0] | 57.0 [51.0, 63.0] | 66.0 [59.0, 74.0] | 64.0 [58.0, 73.0] |
| *Body Mass Index* | 25.6 [22.1, 29.9] | 28.4 [24.7, 33.0] | 25.8 [22.2, 30.1] | 29.2 [25.1, 34.2] | 25.6 [22.1, 29.9] | 28.3 [24.6, 32.9] |
| *Alcohol Use* | | | | | | |
| Current | 1333 (25.4) | 1894 (36.1) | 1187 (26.1) | 5482 (34.3) | 1333 (25.4) | 1413 (26.9) |
| Past | 762 (14.5) | 499 (9.5) | 659 (14.5) | 1272 (8.0) | 762 (14.5) | 666 (12.7) |
| Never | 1137 (21.7) | 1389 (26.5) | 963 (21.2) | 3506 (22.0) | 1137 (21.7) | 1104 (21.0) |
| Unknown | 2018 (38.4) | 1468 (28.0) | 1734 (38.2) | 5697 (35.7) | 2018 (38.4) | 2067 (39.4) |
| *Tobacco Use* | | | | | | |
| Current | 1199 (22.8) | 808 (15.4) | 1095 (24.1) | 3194 (20.0) | 1199 (22.8) | 1264 (24.1) |
| Past | 2227 (42.4) | 1333 (25.4) | 1900 (41.8) | 3015 (18.9) | 2227 (42.4) | 1980 (37.7) |
| Never | 454 (8.6) | 2142 (40.8) | 382 (8.4) | 5387 (33.8) | 454 (8.6) | 528 (10.1) |
| Unknown | 1370 (26.1) | 967 (18.4) | 1166 (25.7) | 4361 (27.3) | 1370 (26.1) | 1478 (28.2) |
| *ADI* | 106.0 [93.8, 118.1] | 101.4 [91.9, 113.1] | 106.3 [93.9, 118.1] | 106.3 [95.3, 119.6] | 106.0 [93.8, 118.1] | 106.0 [95.8, 118.4] |

**Note:** Only variables used in matching/adjustment presented in this table; year of diagnosis/visit suppressed for readability. NHPI = Native Hawaiian/Pacific Islander. ADI = Area Deprivation Index.

**Table S2.** Listing of overall statistically significant and positive associations between point source industrial emissions with incident breast and lung cancers.

| Cancer | Agent | Distance | Type |
| --- | --- | --- | --- |
| Breast | Benzene | 20 | IDW (1) |
|  | Chromium | 5 | Presence (1, 2, 3, 4, 5), Number (3, 4, 5) |
|  |  | 10 | Number (1, 2, 3, 4, 5) |
|  |  | 20 | Presence (1, 2, 3, 4, 5), Number (1, 2, 3, 4, 5) |
|  | Ethylene Oxide | 5 | Presence (1, 2, 3, 4), Number (1, 2, 3, 4), IDW (1, 2, 3, 4, 5) |
|  |  | 10 | Presence (1, 2, 3, 4), Number (1, 2, 3, 4) |
|  |  | 20 | Presence (1, 2, 3, 4), Number (1, 2, 3, 4, 5) |
|  | Formaldehyde | 5 | Presence (4, 5) |
|  |  | 10 | Presence (2, 3, 4, 5), Number (4, 5) |
|  |  | 20 | Presence (1, 2, 3, 4, 5), Number (1, 2, 3, 4, 5) |
|  | Nickel | 5 | Presence (2, 3), Number (2, 3, 4, 5) |
|  |  | 10 | Number (1, 2, 3, 4, 5) |
| Lung | Benzene | 5 | IDW (3, 4, 5) |
|  |  | 10 | IDW (1, 2, 3, 4, 5) |
|  |  | 20 | IDW (1) |
|  | Chromium | 5 | Presence (1, 2, 3, 4, 5), Number (1, 2, 3, 4, 5) |
|  |  | 10 | Presence (1, 2, 3, 4, 5), Number (1, 2, 3, 4, 5) |
|  |  | 20 | Presence (1, 2, 3, 4, 5), Number (1, 2, 3, 4, 5) |
|  | Cobalt | 10 | Presence (1, 2, 3, 4, 5), Number (1, 2, 3, 4, 5) |
|  | Ethylene Oxide | 5 | Presence (1, 2, 3, 4), Number (1, 2, 3, 4, 5), IDW (2, 3, 4, 5) |
|  |  | 10 | Number (1, 2, 3, 4, 5) |
|  |  | 20 | Number (1, 2, 3, 4, 5) |
|  | Formaldehyde | 5 | Presence (4, 5), Number (5) |
|  |  | 10 | Presence (1, 2, 3, 4, 5), Number (1, 2, 3, 4, 5), IDW (4, 5) |
|  |  | 20 | Presence (1, 2, 3, 4, 5), Number (1, 2, 3, 4, 5), IDW (1, 2, 3, 4, 5) |
|  | Nickel | 5 | Presence (3, 5), Number (1, 2, 3, 4, 5) |
|  |  | 10 | Number (1, 2, 3, 4, 5) |
|  |  | 20 | Number (1, 2, 3, 4, 5) |

**Note:** In the Type column, the numbers in parentheses denote cumulative year lags for that combination of agent, distance, and type (presence, number, or IDW). Significance determined by Bonferroni-adjusted p-values < 0.01 from conditional logistic regression and positive odds ratios for all three matched analyses. Significant inverse associations described in text and omitted from table due to very small numbers. IDW = Inverse-distance weighted emission quantity.

**Table S3.** Listing of overall statistically significant and positive associations between point source industrial emissions with incident breast and lung cancers in the sensitivity analysis.

| Cancer | Agent | Distance | Type |
| --- | --- | --- | --- |
| Breast | Benzene | 20 | IDW (1, 2, 3, 4, 5) |
|  | Chromium | 5 | Presence (1, 2, 3, 4, 5), Number (2, 3, 4, 5) |
|  |  | 10 | Number (1, 2, 3, 4, 5) |
|  |  | 20 | Presence (1, 3, 4, 5), Number (1, 2, 3, 4, 5) |
|  | Formaldehyde | 20 | Presence (5) |
|  | Nickel | 5 | Number (2, 3, 4, 5) |
|  |  | 10 | Number (2, 3, 4, 5) |
| Lung | Benzene | 20 | IDW (4, 5) |
|  | Chromium | 20 | Presence (1, 5), Number (1, 2, 3, 4, 5) |
|  | Formaldehyde | 20 | Presence (4, 5), Number (5), IDW (5) |

**Note:** In the Type column, the numbers in parentheses denote cumulative year lags for that combination of agent, distance, and type (presence, number, or IDW). Significance determined by Bonferroni-adjusted p-values < 0.01 from conditional logistic regression and positive odds ratios for all three matched analyses in sensitivity analysis restricting both cancer types to never-smokers. IDW = Inverse-distance weighted emission quantity.

**Table S4.** Proportion of statistically significant and positive associations between point source industrial emissions with incident breast and lung cancers by matching approach, sensitivity analysis.

| Agent | Breast cancer | | | Lung cancer | | |
| --- | --- | --- | --- | --- | --- | --- |
|  | Nearest-Neighbor | Exact | Optimal | Nearest-Neighbor | Exact | Optimal |
| Ethylene Oxide | 23 | 0 | 8 | 0 | 0 | 0 |
| Formaldehyde | 25 | 7 | 10 | 17 | 35 | 7 |
| Benzene | 33 | 8 | 8 | 8 | 10 | 3 |
| Chromium | 52 | 38 | 50 | 33 | 52 | 12 |
| Nickel | 57 | 15 | 25 | 8 | 18 | 2 |
| Arsenic | 30 | 0 | 0 | 0 | 0 | 0 |
| Cobalt | 49 | 0 | 0 | 11 | 0 | 0 |
| Hydrazine | 0 | 8 | 0 | 0 | 0 | 0 |
| Antimony | 4 | 0 | 0 | 0 | 0 | 0 |

**Note**: Numbers in the table are given as percentages. Significance is based on Bonferroni-adjusted p-values. Sensitivity analysis restricted both cancer types to never-smokers. Beryllium and cadmium are omitted from the table due to an absence of any significant Bonferroni-adjusted p-values for any method or cancer.

**Figure S1.** Distribution of number of antimony facilities, breast cancer analysis.

**
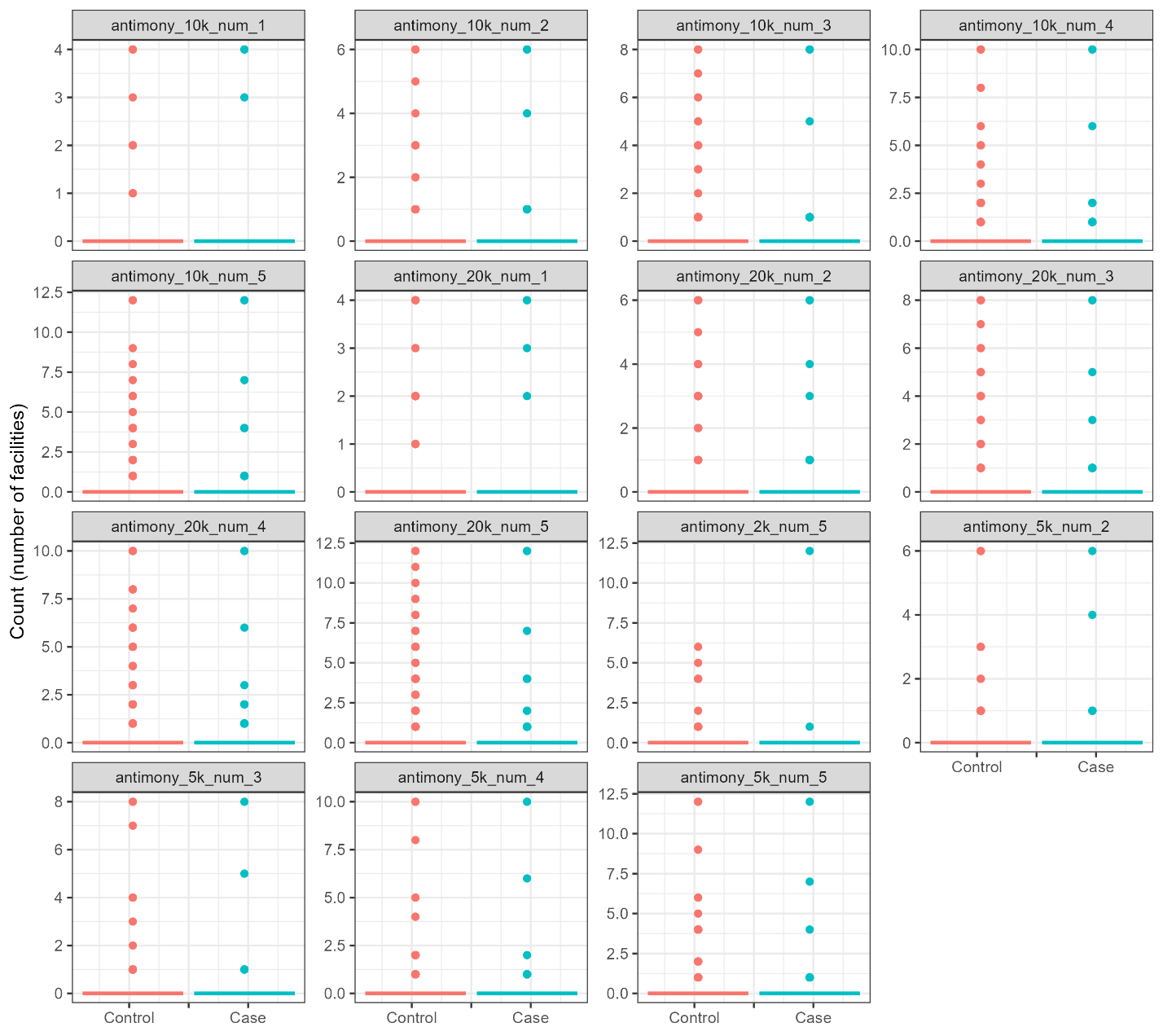
**

**Figure S2.** Distribution of number of arsenic facilities, breast cancer analysis.


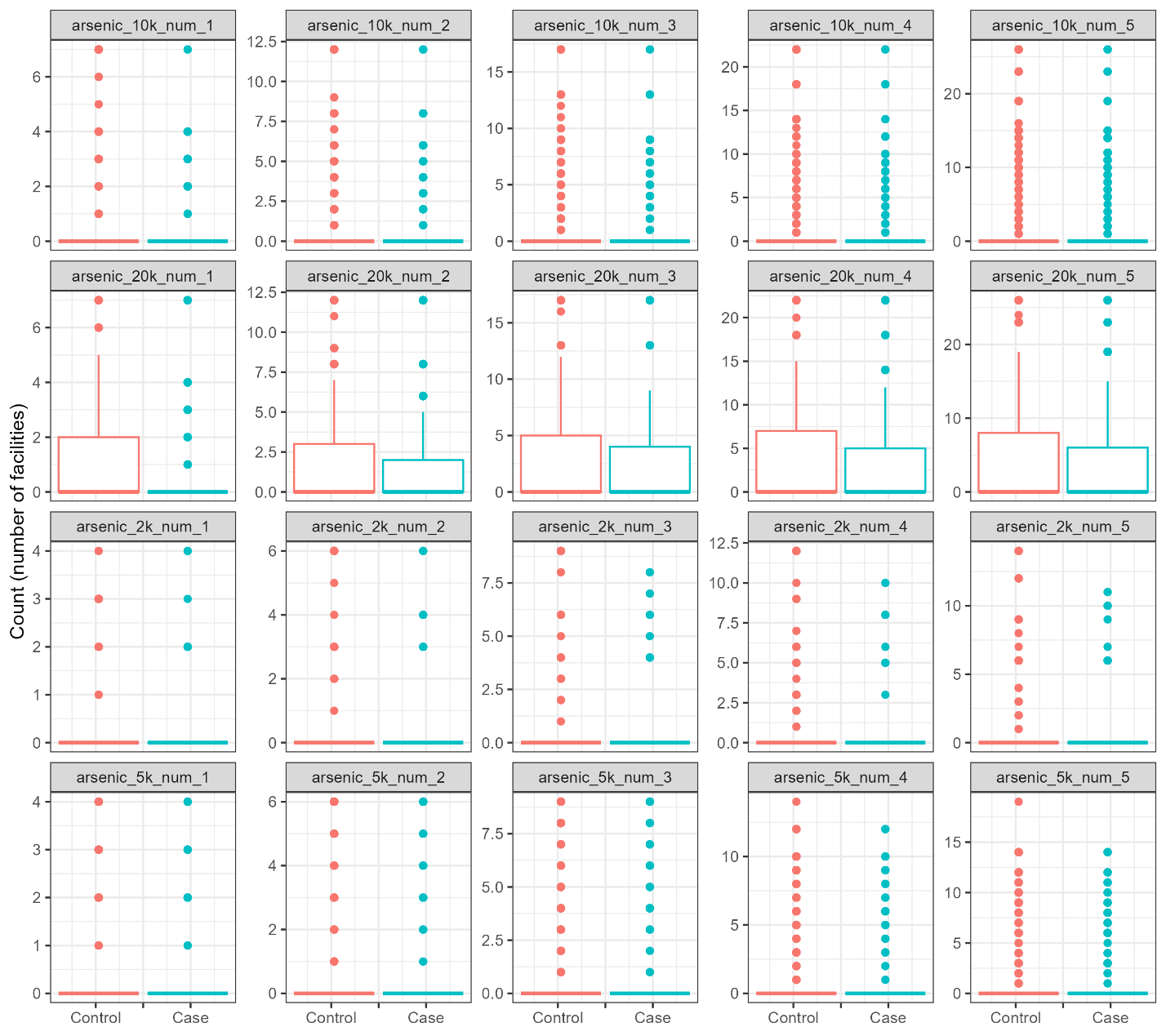


**Figure S3.** Distribution of number of benzene facilities, breast cancer analysis.

**
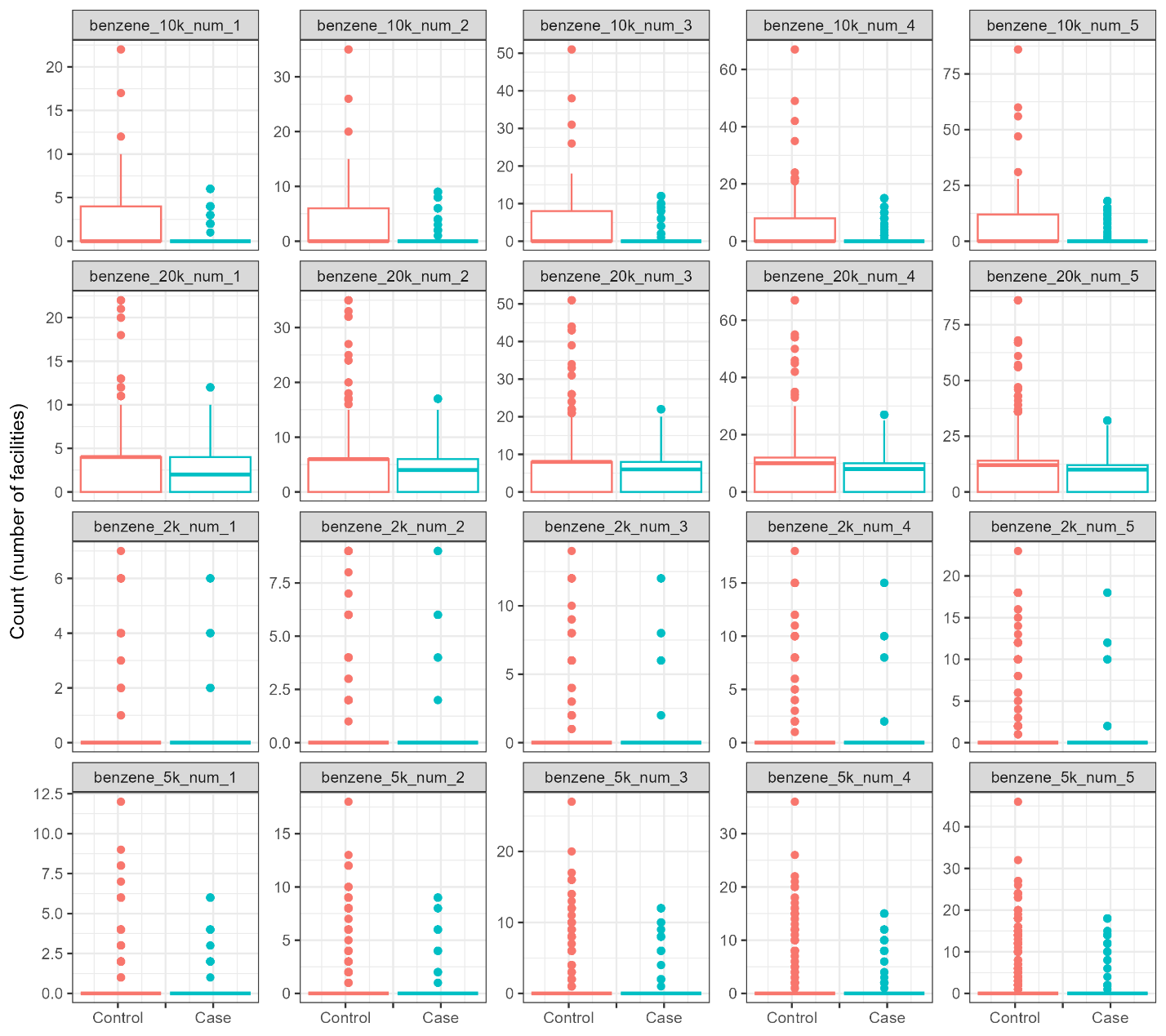
**

**Figure S4.** Distribution of number of beryllium facilities, breast cancer analysis.

**
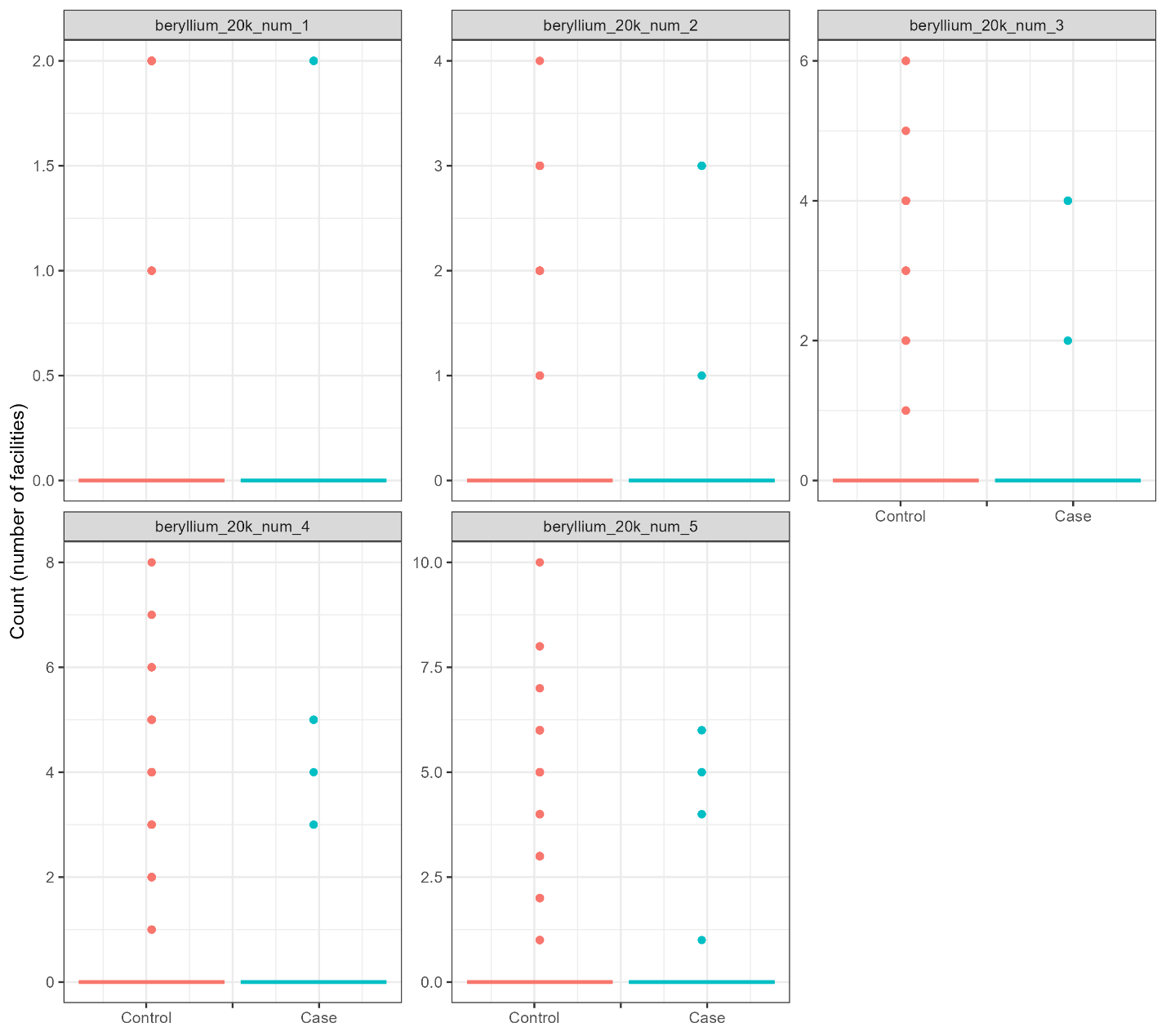
**

**Figure S5.** Distribution of number of cadmium facilities, breast cancer analysis.

**
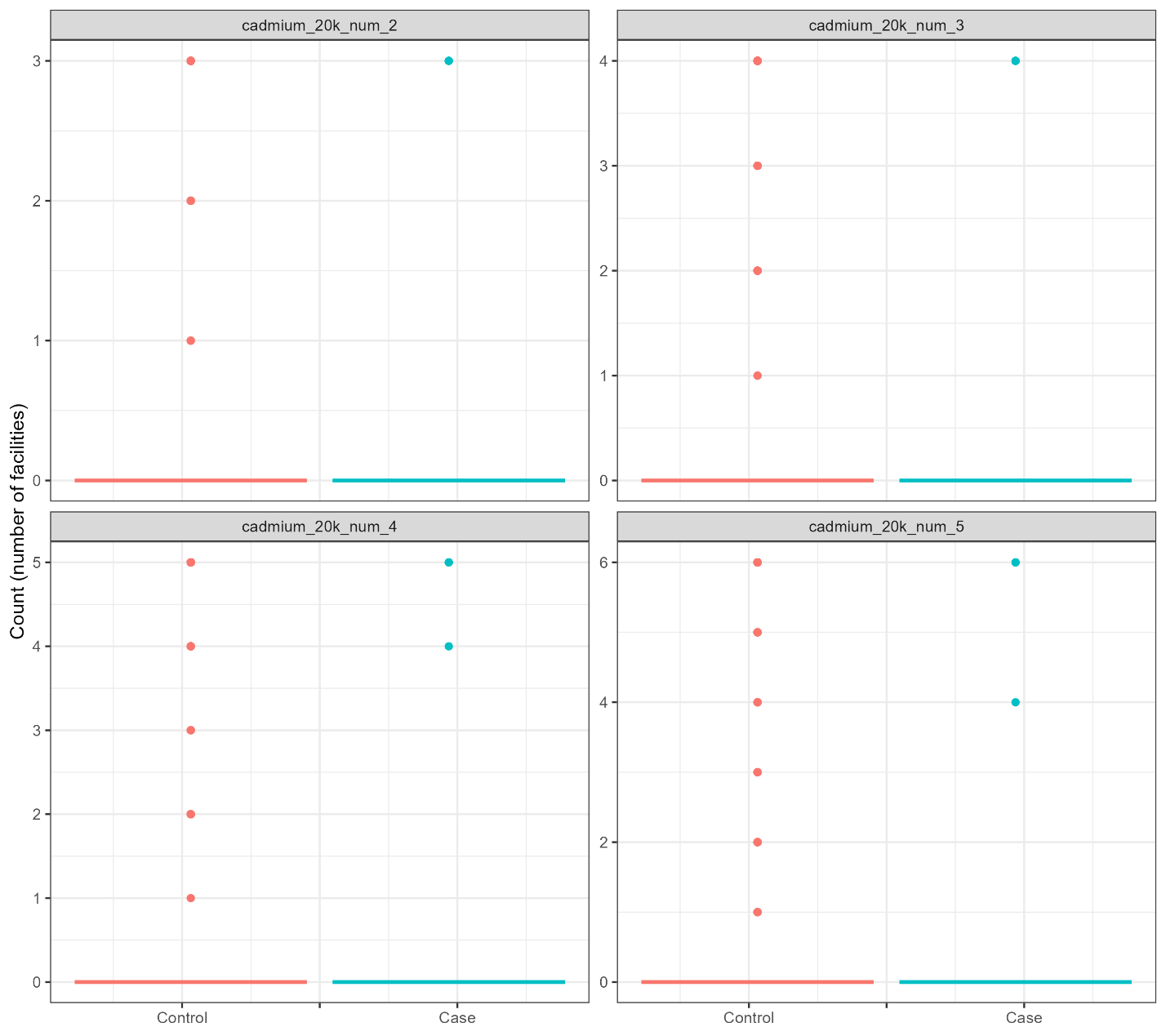
**

**Figure S6.** Distribution of number of chromium facilities, breast cancer analysis.

**
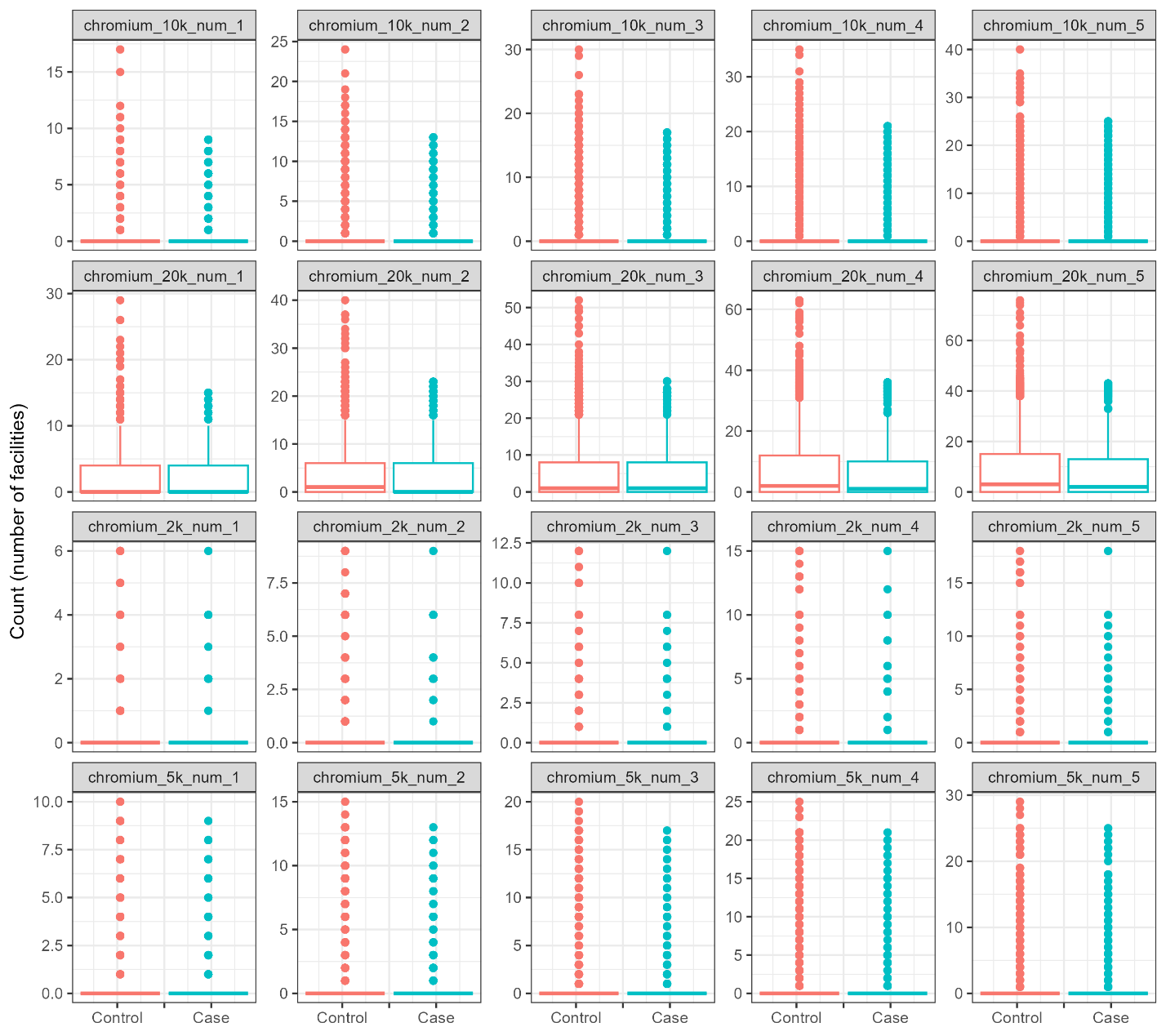
**

**Figure S7.** Distribution of number of cobalt facilities, breast cancer analysis.

**
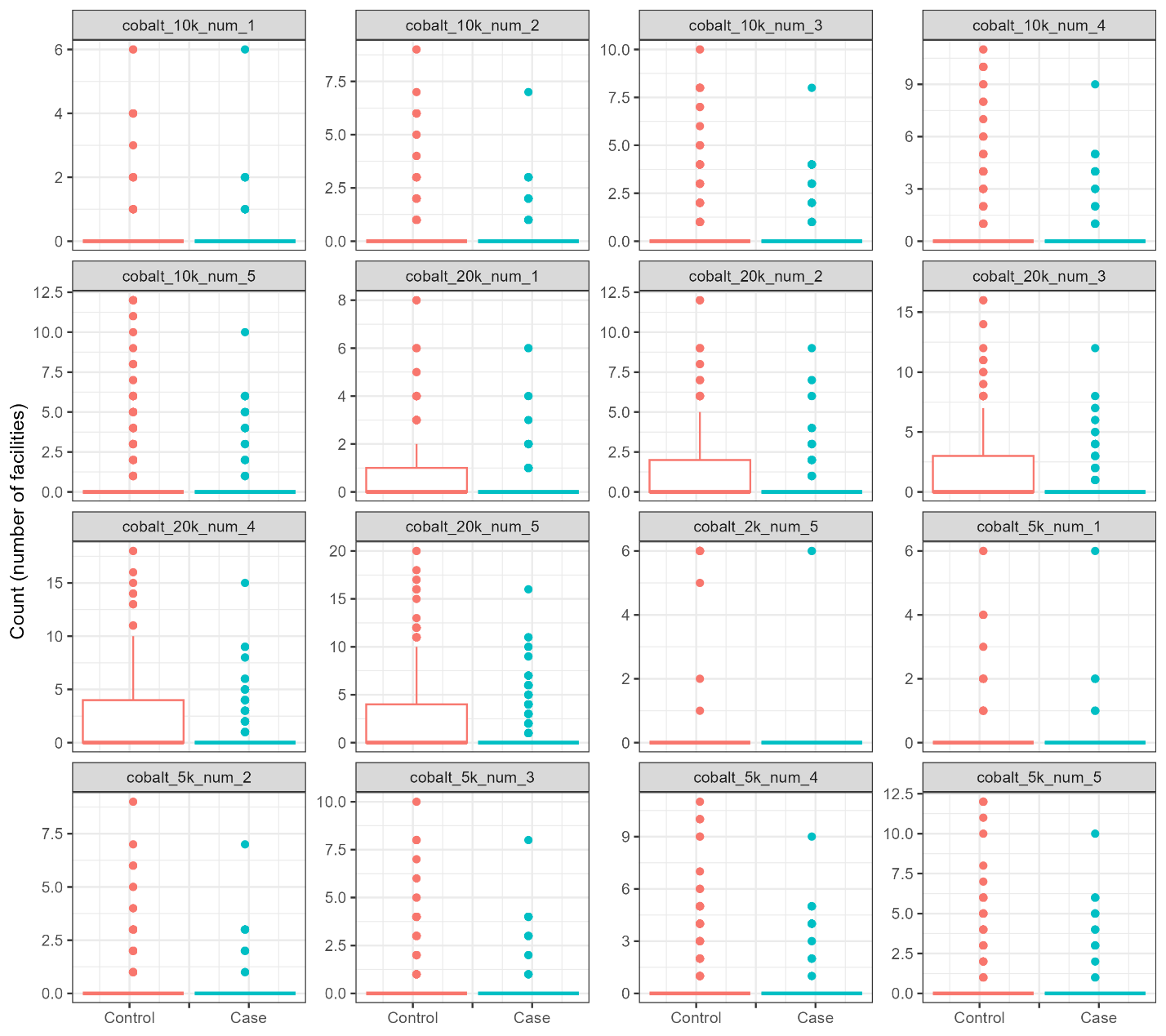
**

**Figure S8.** Distribution of number of ethylene oxide facilities, breast cancer analysis.

**
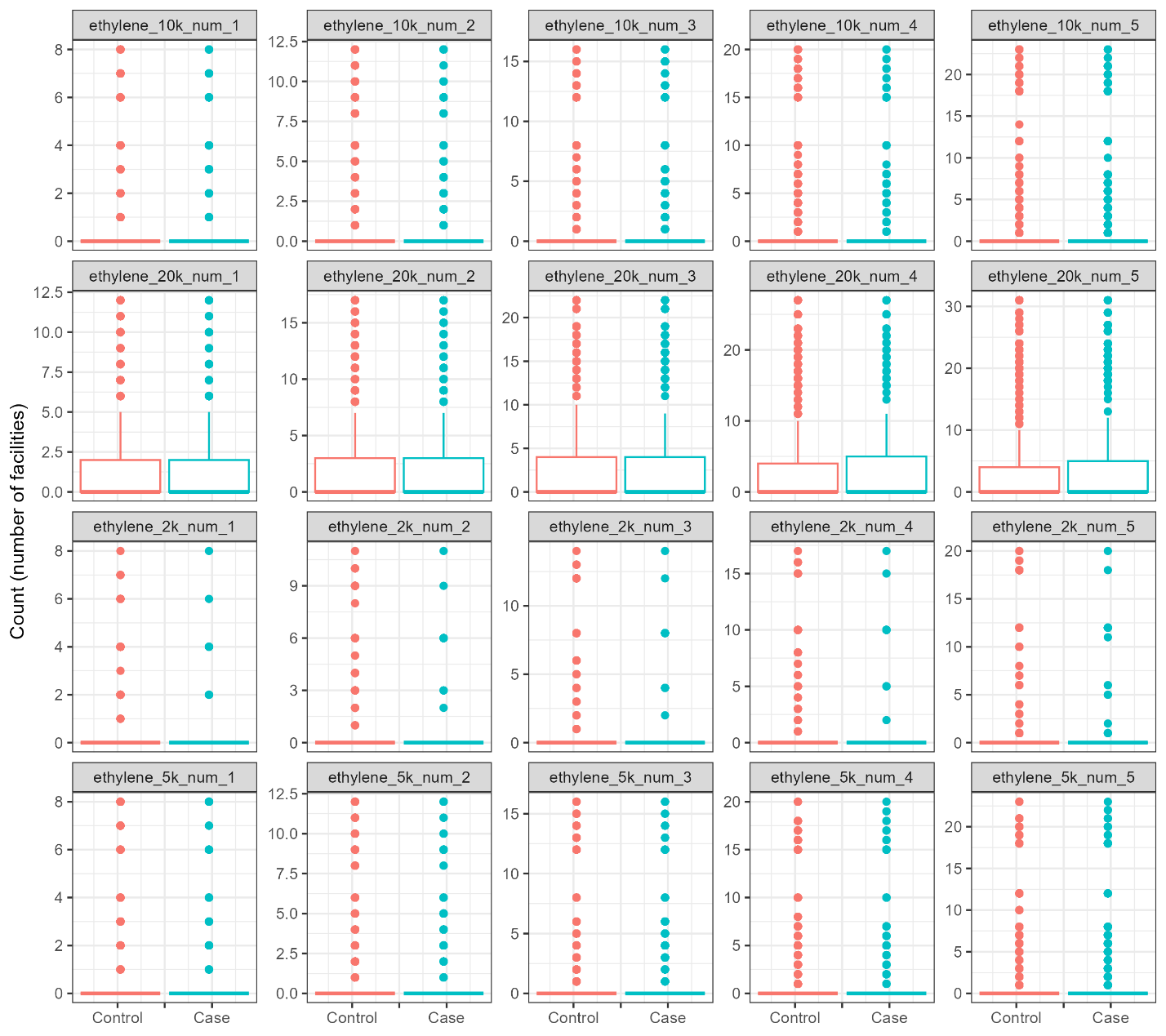
**

**Figure S9.** Distribution of number of formaldehyde facilities, breast cancer analysis.

**
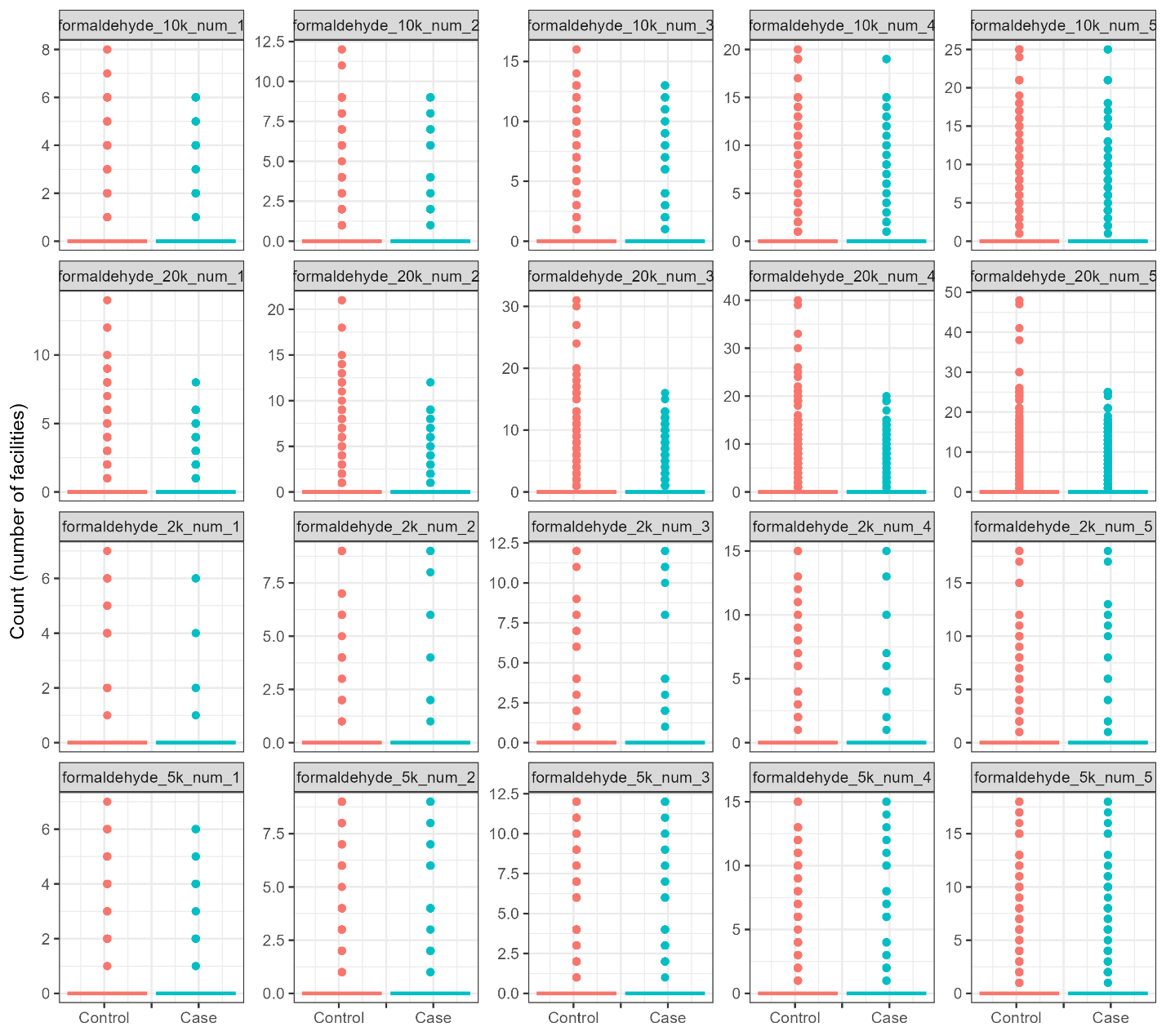
**

**Figure S10.** Distribution of number of hydrazine facilities, breast cancer analysis.

**
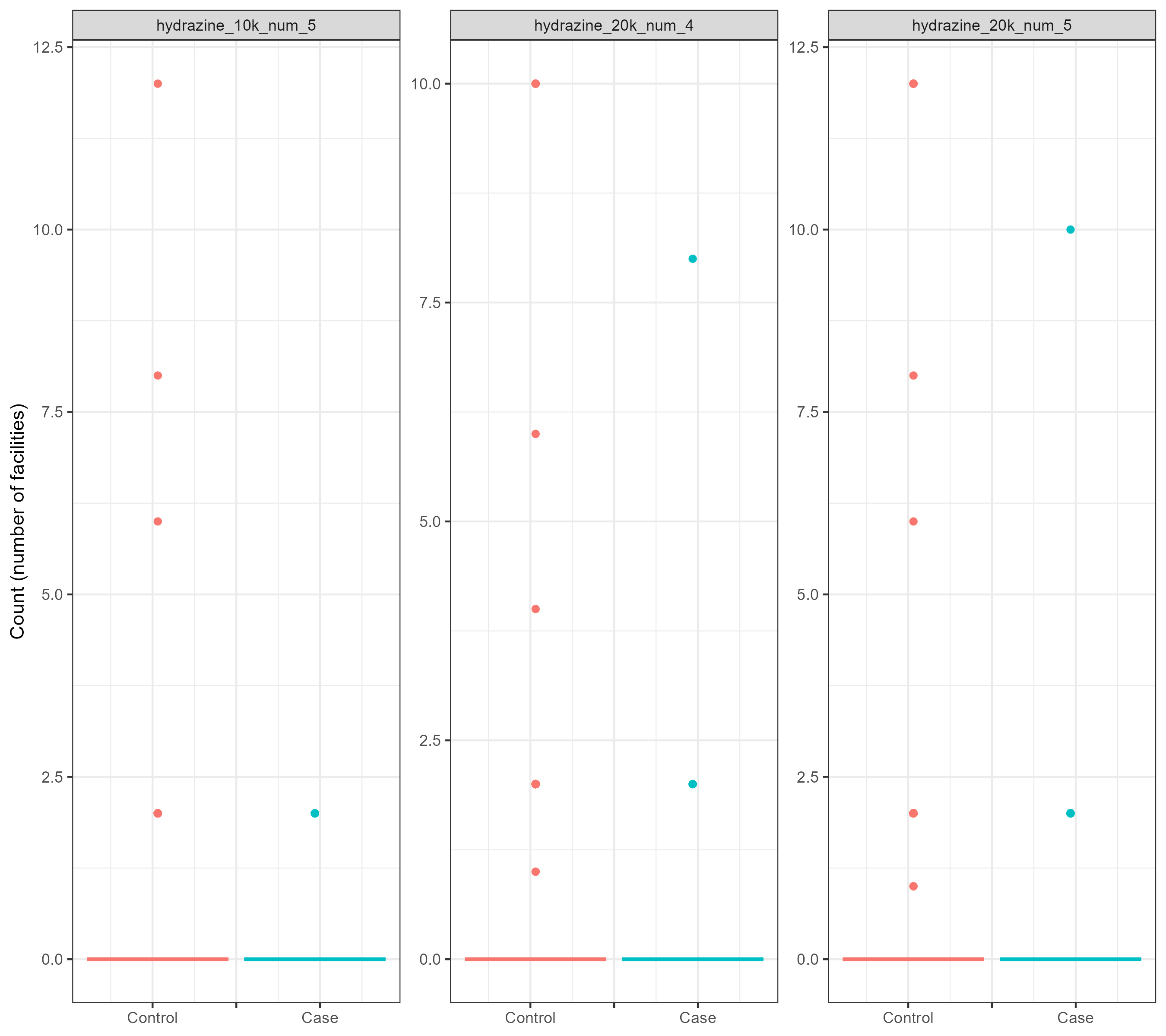
**

**Figure S11.** Distribution of number of nickel facilities, breast cancer analysis.

**
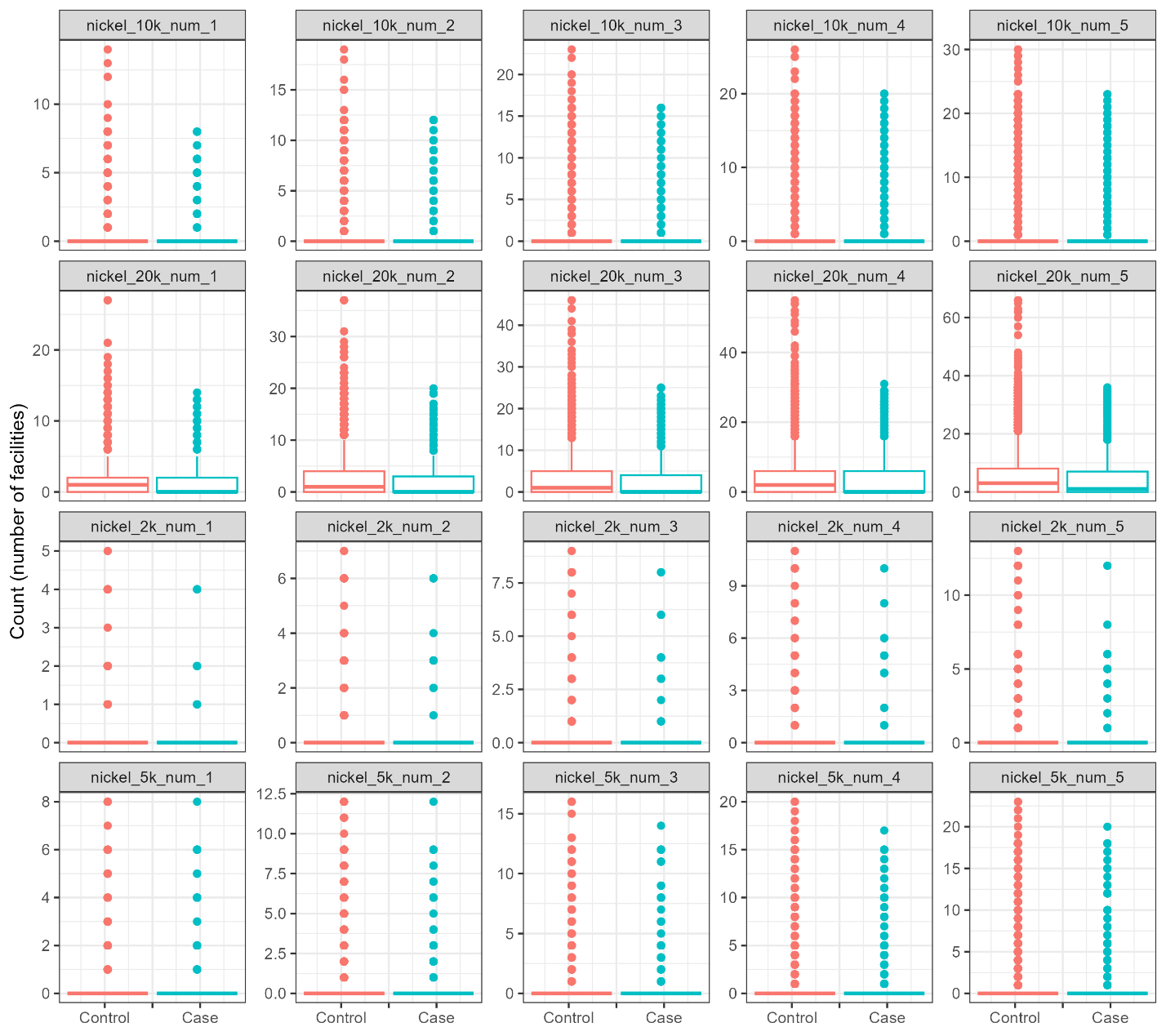
**

**Figure S12.** Distribution of number of antimony facilities, lung cancer analysis.

**
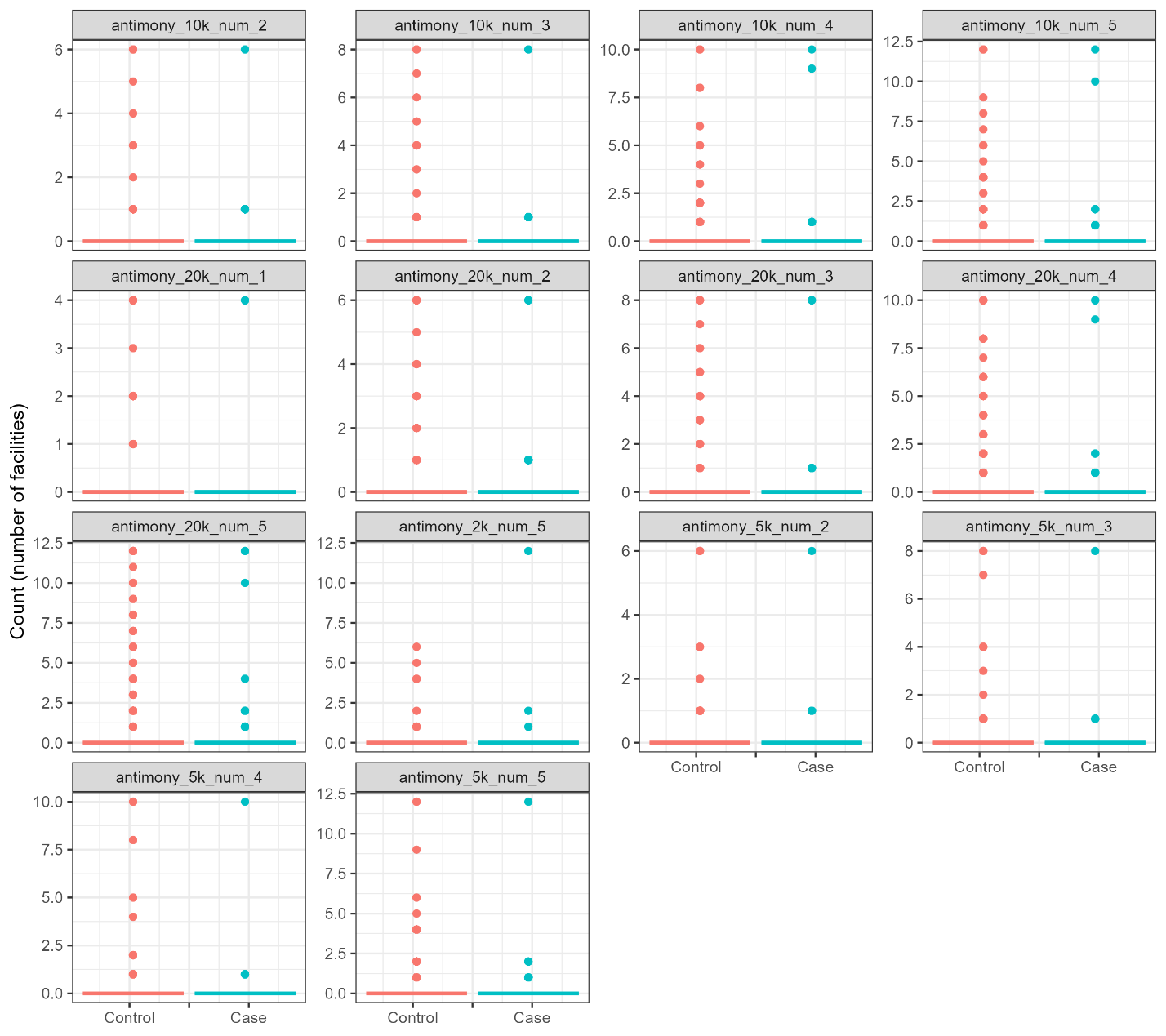
**

**Figure S13.** Distribution of number of arsenic facilities, lung cancer analysis.

**
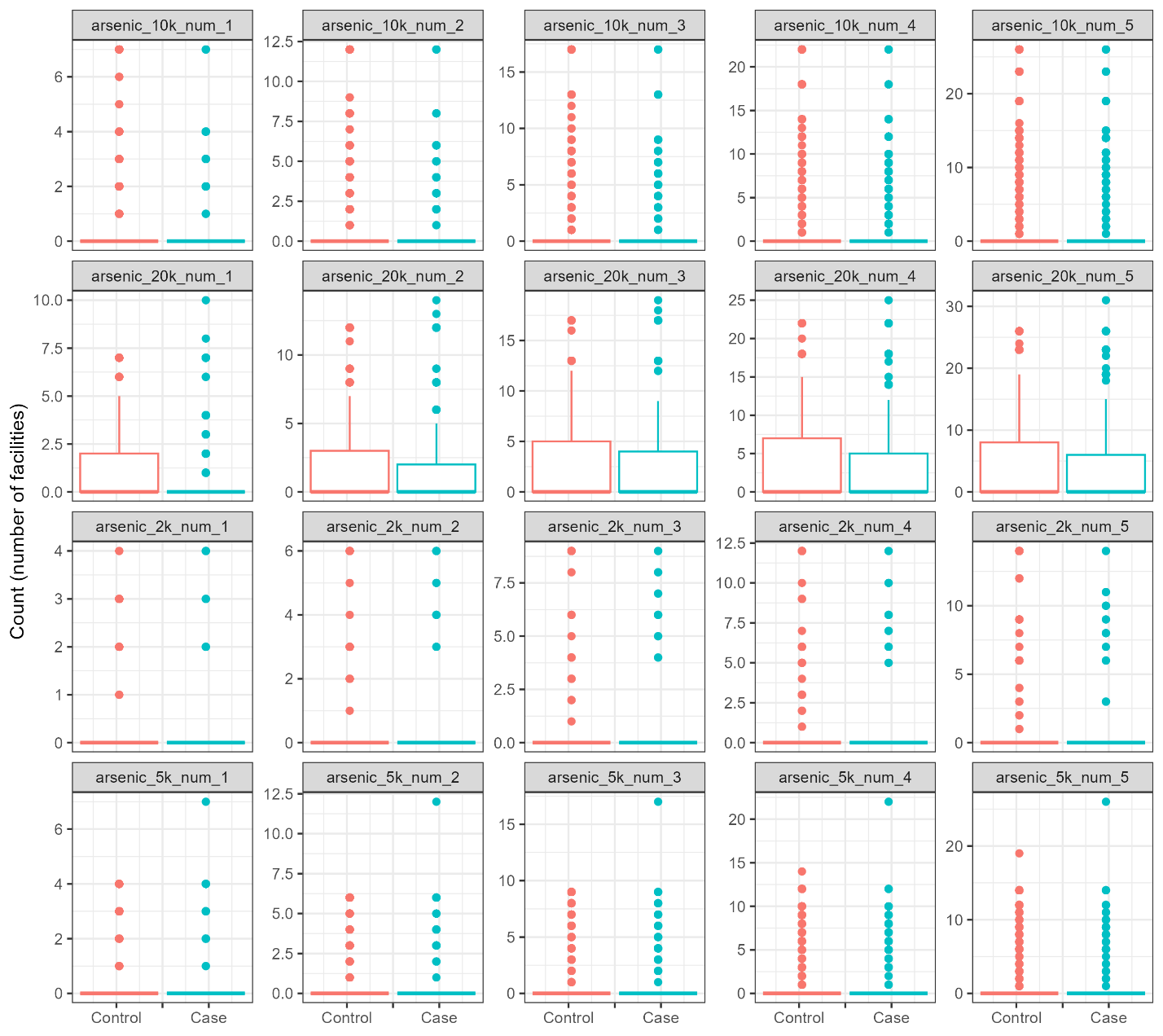
**

**Figure S14.** Distribution of number of benzene facilities, lung cancer analysis.

**
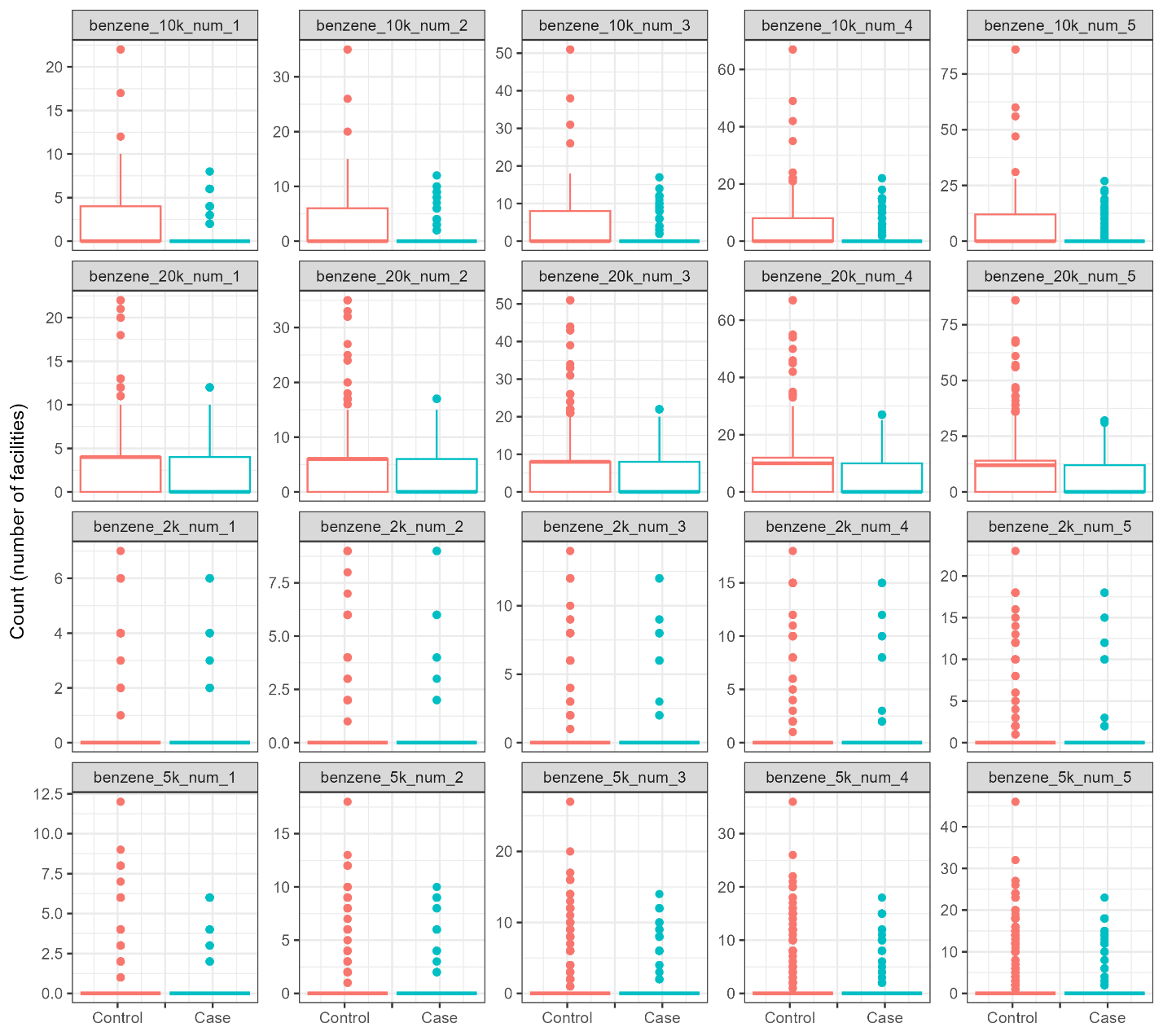
**

**Figure S15.** Distribution of number of beryllium facilities, lung cancer analysis.

**
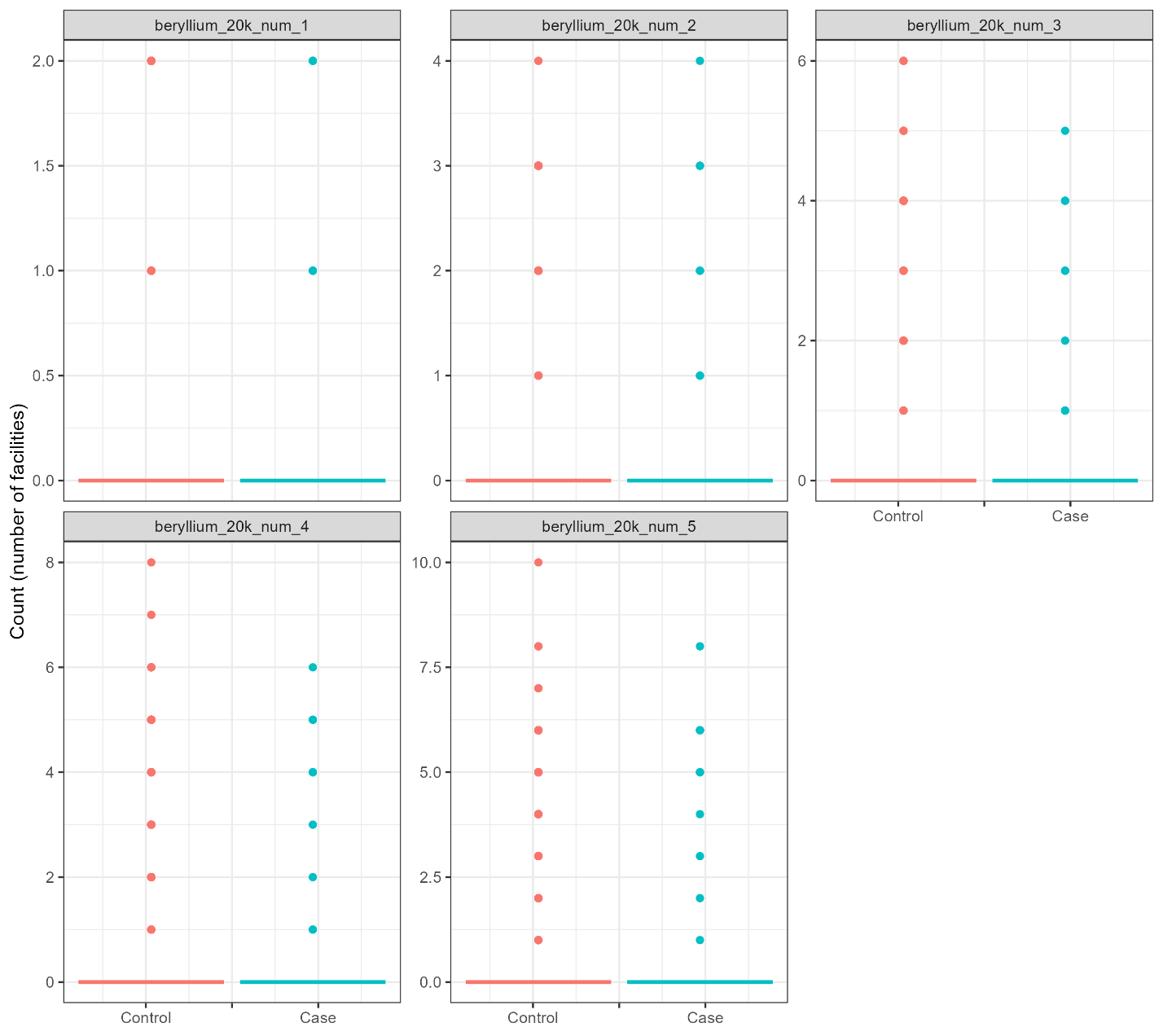
**

**Figure S16.** Distribution of number of cadmium facilities, lung cancer analysis.

**
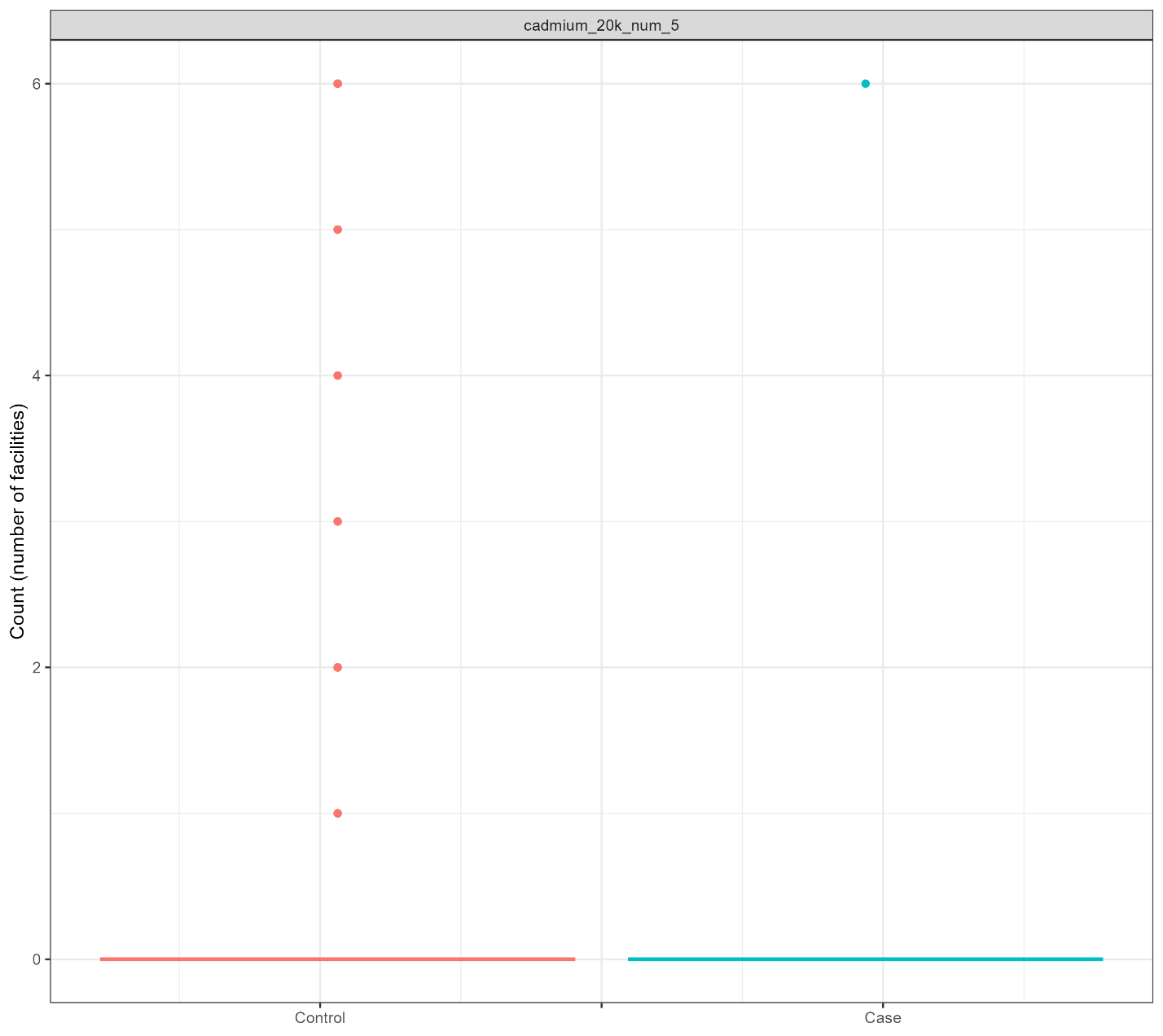
**

**Figure S17.** Distribution of number of chromium facilities, lung cancer analysis.

**
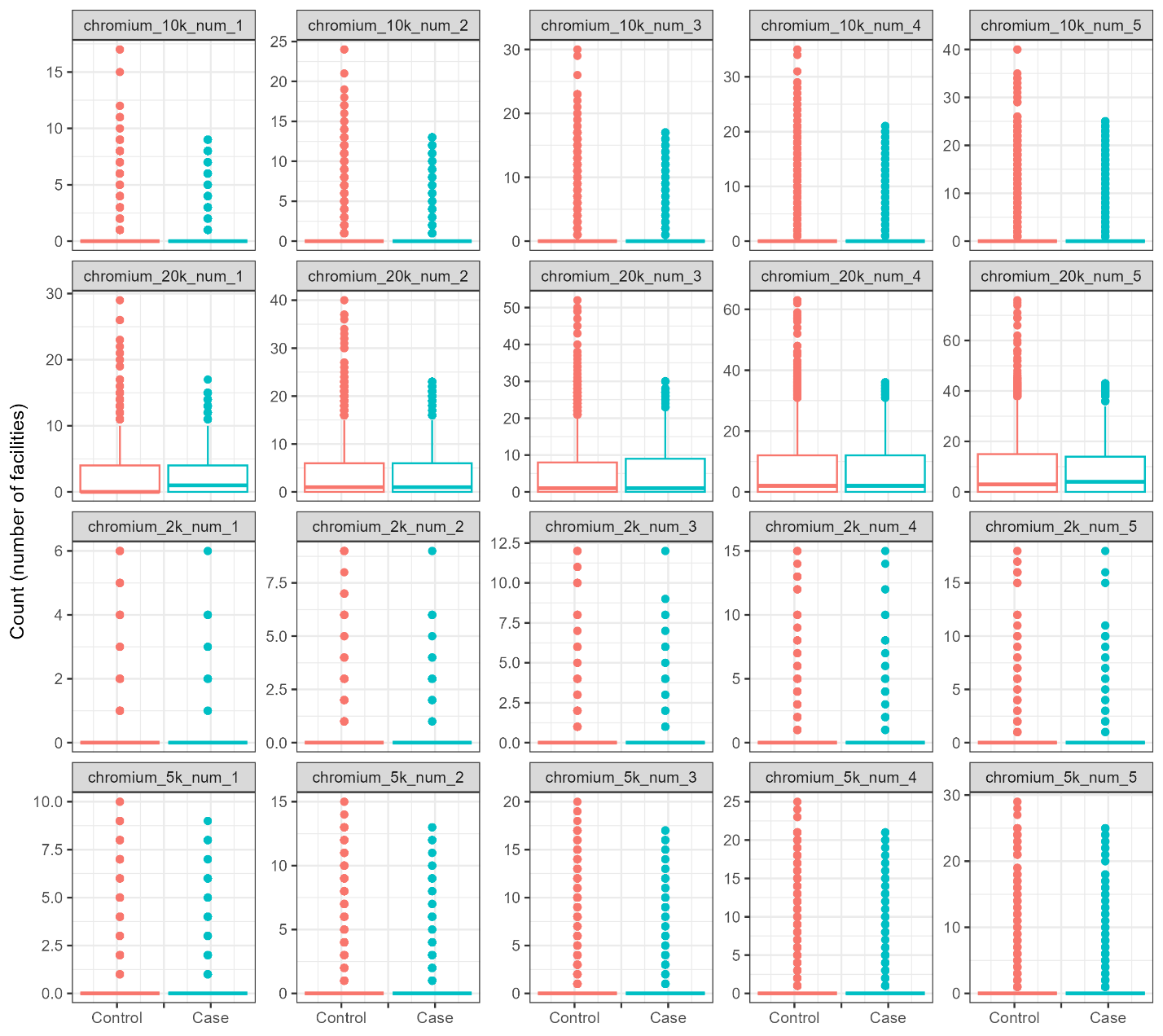
**

**Figure S18.** Distribution of number of cobalt facilities, lung cancer analysis.

**
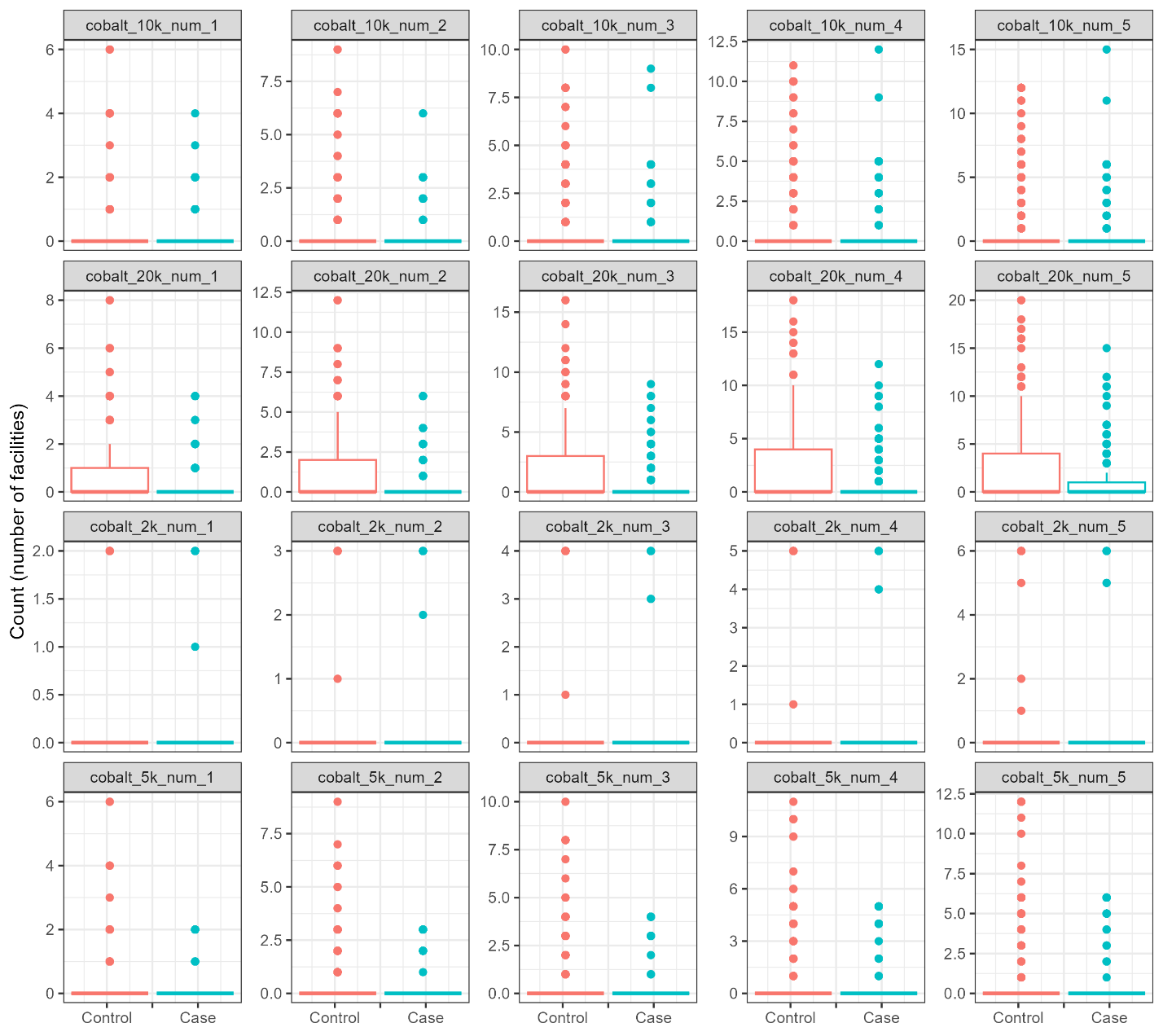
**

**Figure S19.** Distribution of number of ethylene oxide facilities, lung cancer analysis.

**
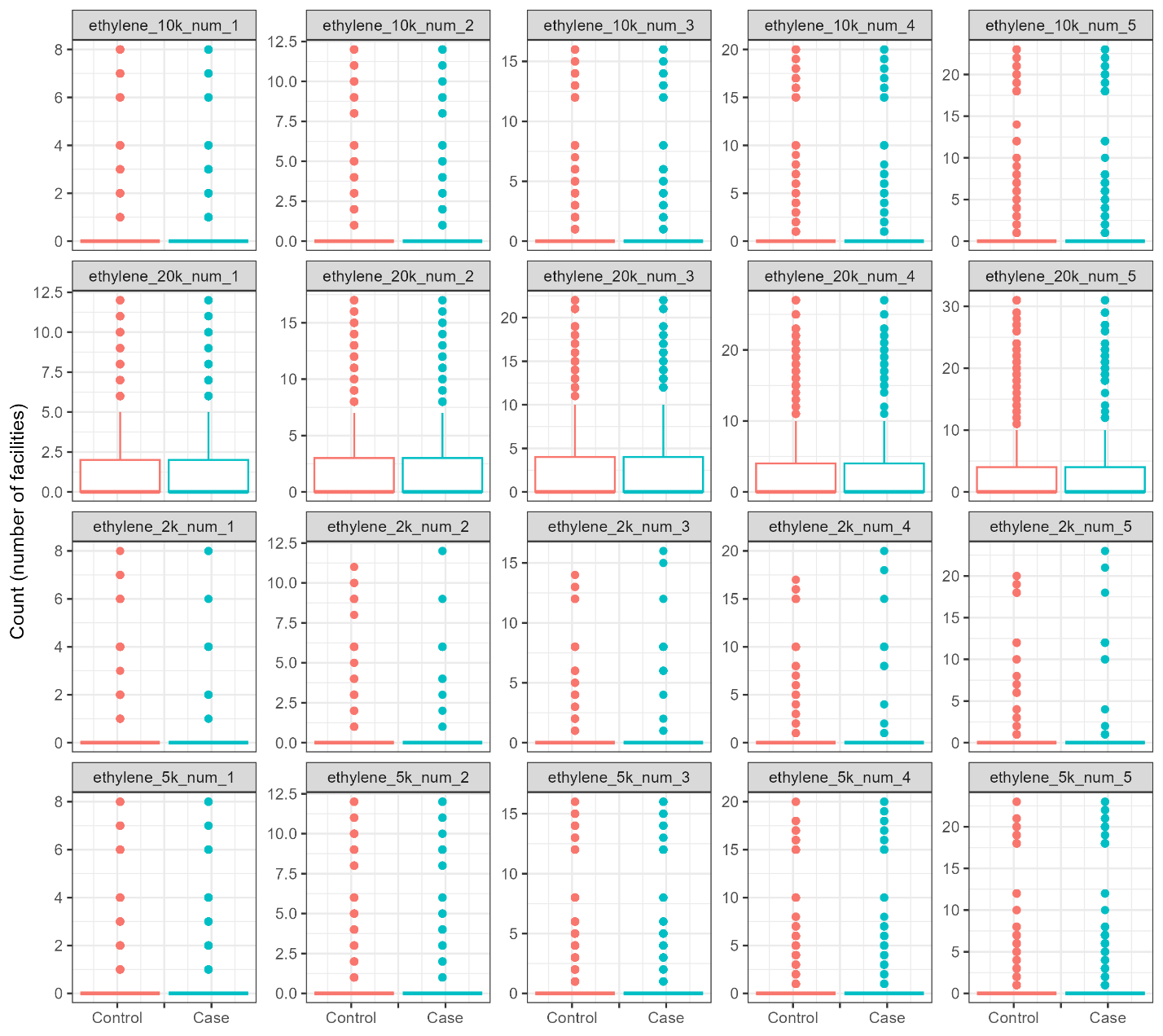
**

**Figure S20.** Distribution of number of formaldehyde facilities, lung cancer analysis.

**
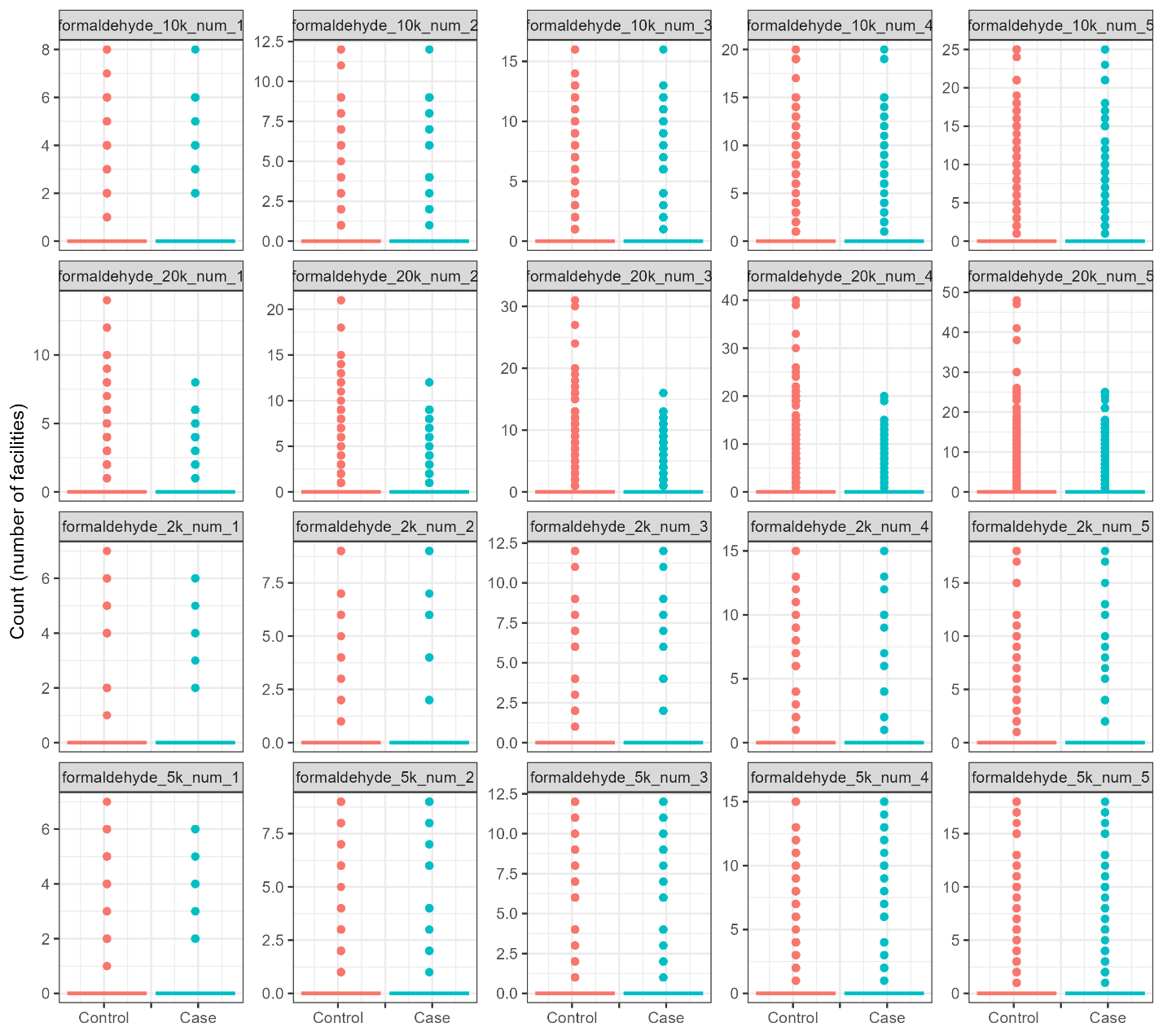
**

**Figure S21.** Distribution of number of hydrazine facilities, lung cancer analysis.


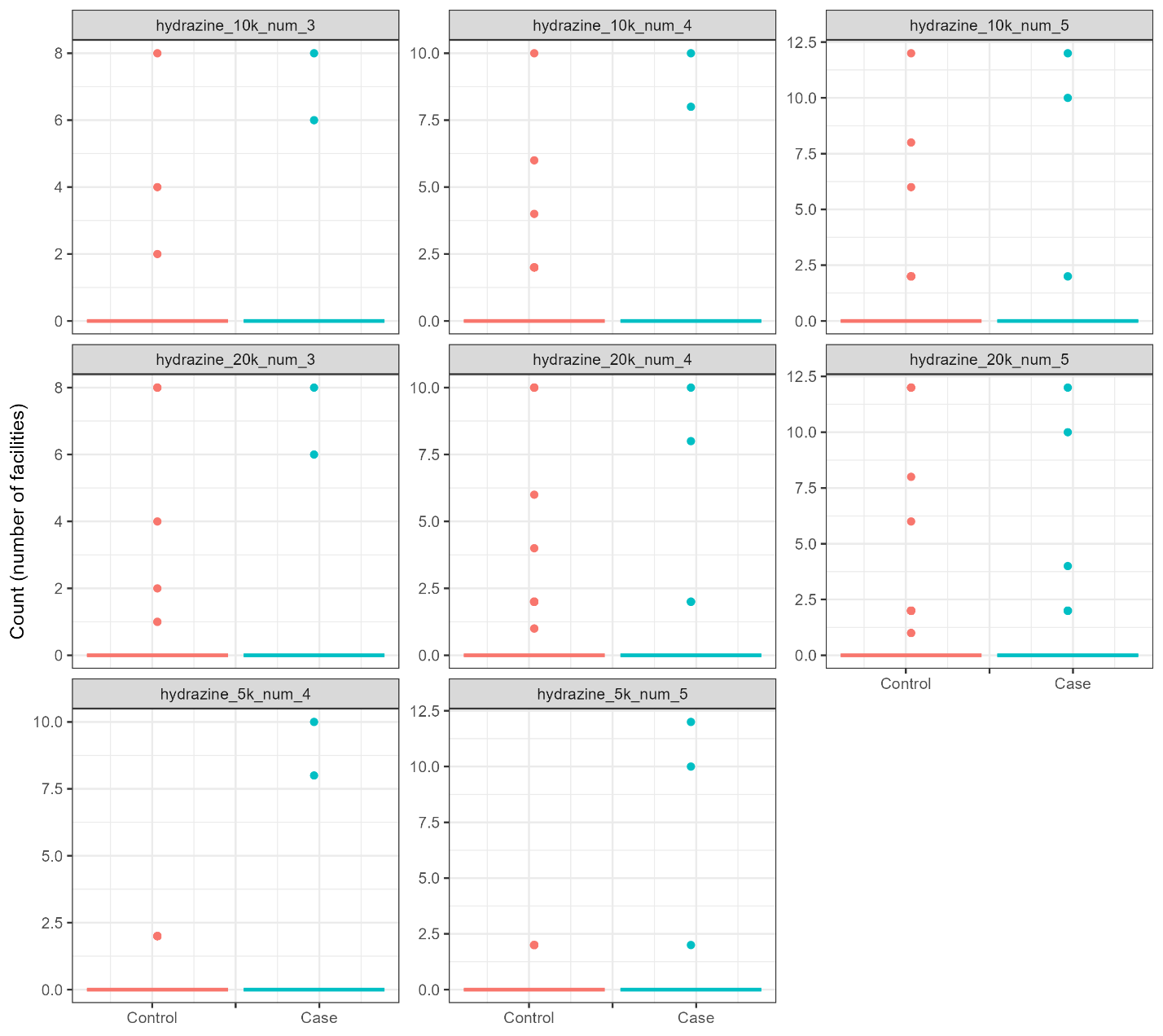


**Figure S22.** Distribution of number of nickel facilities, lung cancer analysis.

**
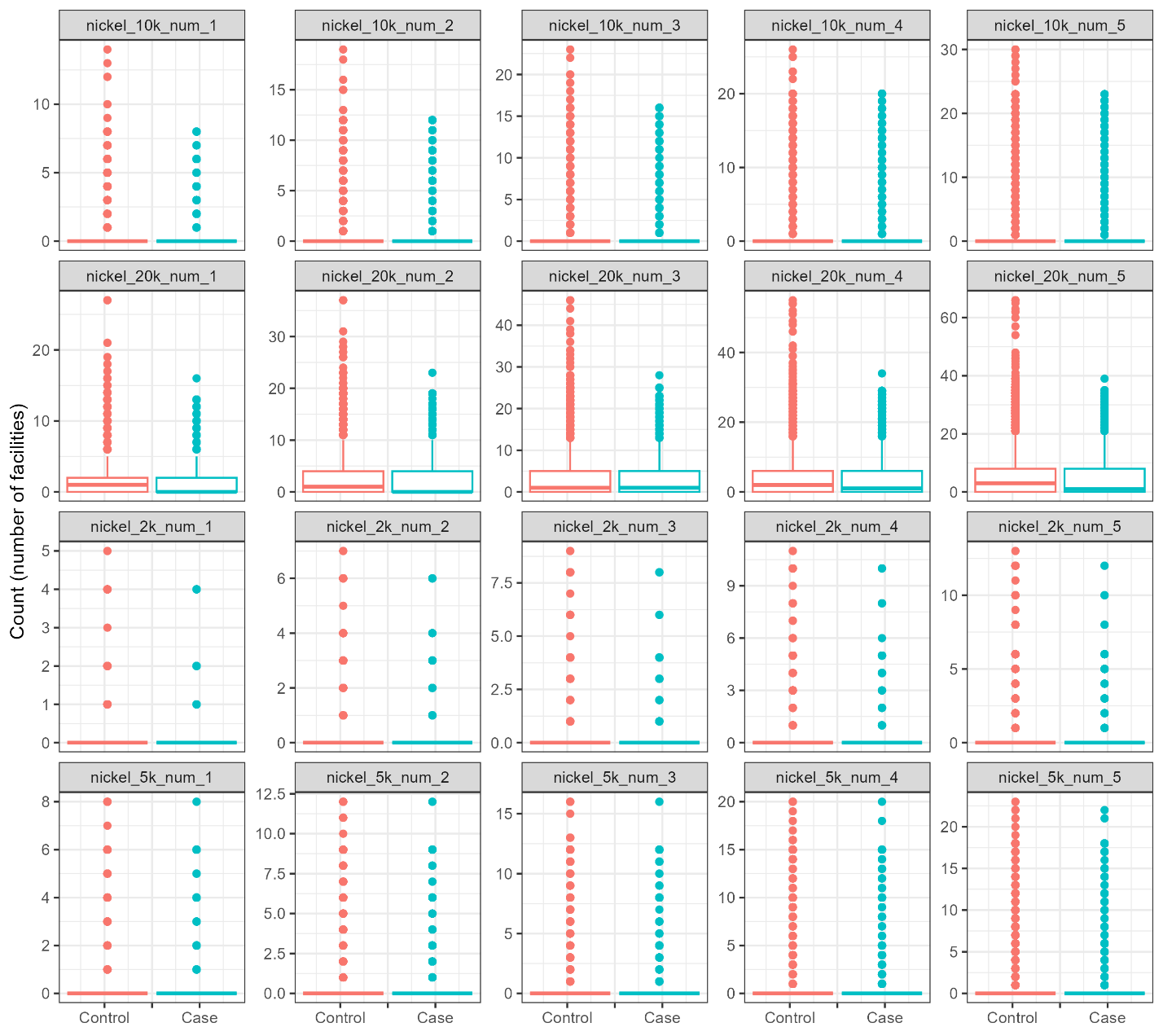
**

**Figure S23.** Volcano plot for point source industrial emissions and breast cancer by agent and matching analysis.

**
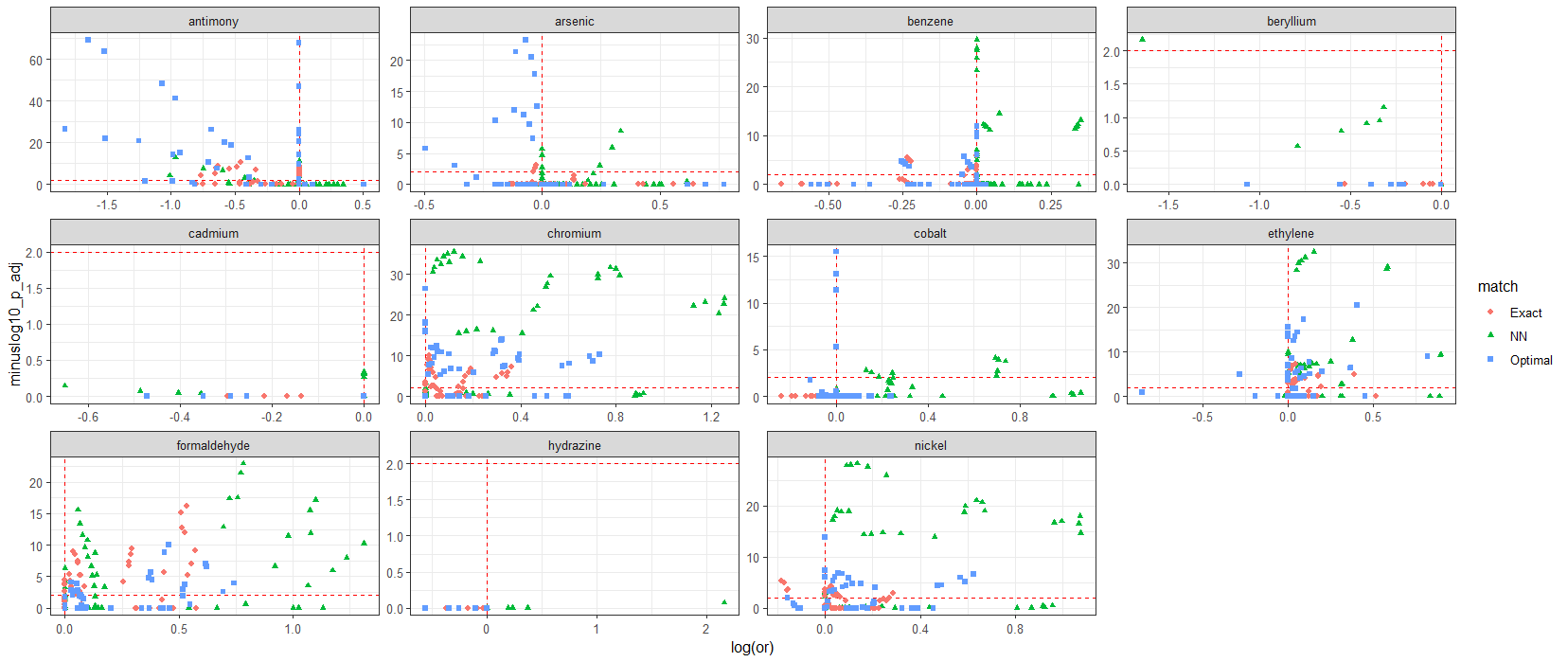
**

**Note:** In the volcano plot, the x-axis is the logarithm of the odds ratio estimated by the conditional logistic regression model, and the y-axis is the negative logarithm of the Bonferroni-adjusted p-value for the coefficient. As such, higher values on the y-axis denote smaller p-values. The red horizontal dashed line represents the significance threshold (0.01), and the red vertical line represents a logarithm of the odds ratio equal to zero.

**Figure S24.** Volcano plot for point source industrial emissions and lung cancer by agent and matching analysis.

**
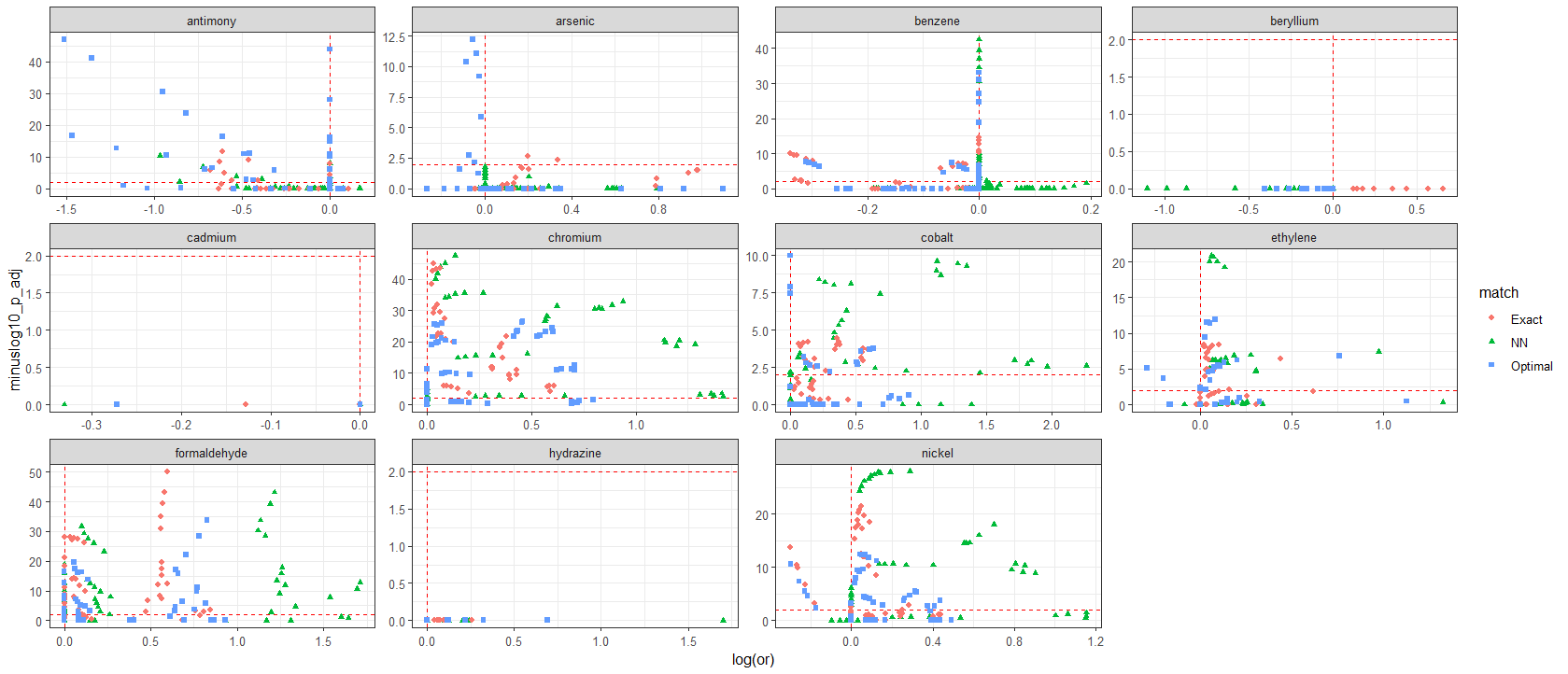
**

**Note:** In the volcano plot, the x-axis is the logarithm of the odds ratio estimated by the conditional logistic regression model, and the y-axis is the negative logarithm of the Bonferroni-adjusted p-value for the coefficient. As such, higher values on the y-axis denote smaller p-values. The red horizontal dashed line represents the significance threshold (0.01), and the red vertical line represents a logarithm of the odds ratio equal to zero.

**Figure S25.** Volcano plot for point source industrial emissions and breast cancer, by matching analysis, sensitivity analysis.


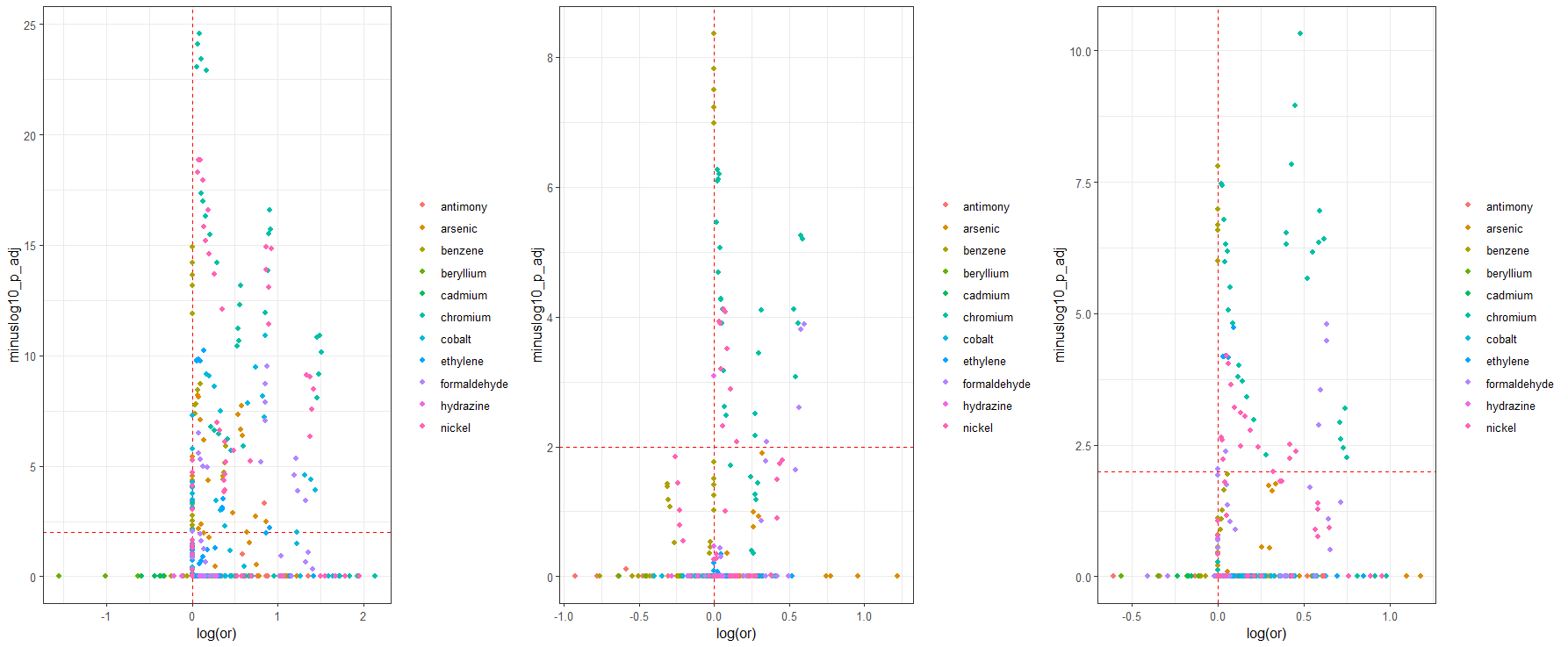


**Note:** Plot panels are from nearest neighbor, exact, and optimal matching from left to right. In the volcano plot, the x-axis is the logarithm of the odds ratio estimated by the conditional logistic regression model, and the y-axis is the negative logarithm of the Bonferroni-adjusted p-value for the coefficient. As such, higher values on the y-axis denote smaller p-values. The red horizontal dashed line represents the significance threshold (0.01), and the red vertical line represents a logarithm of the odds ratio equal to zero. Sensitivity analysis restricted breast cancer cases to never-smokers.

**Figure S26.** Volcano plot for point source industrial emissions and lung cancer, by matching analysis, sensitivity analysis.


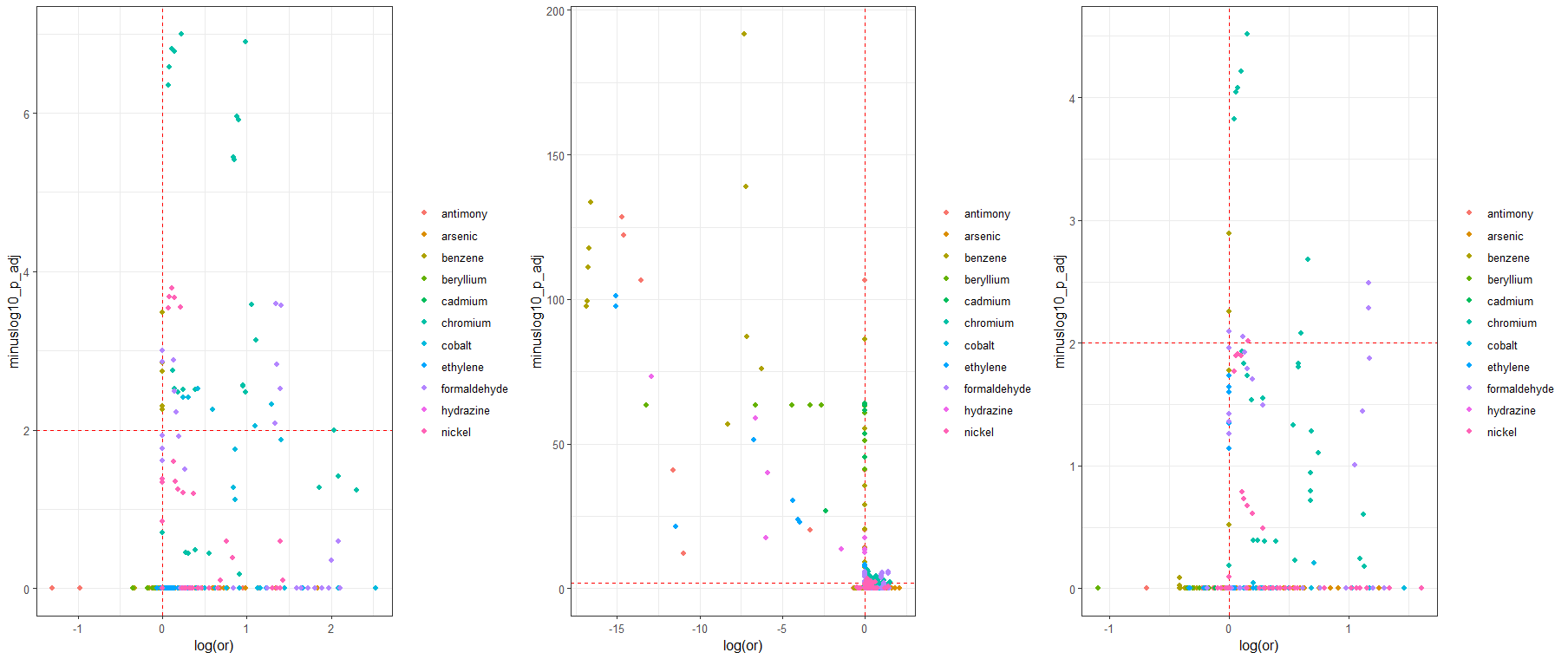


**Note:** Plot panels are from nearest neighbor, exact, and optimal matching from left to right. In the volcano plot, the x-axis is the logarithm of the odds ratio estimated by the conditional logistic regression model, and the y-axis is the negative logarithm of the Bonferroni-adjusted p-value for the coefficient. As such, higher values on the y-axis denote smaller p-values. The red horizontal dashed line represents the significance threshold (0.01), and the red vertical line represents a logarithm of the odds ratio equal to zero. Sensitivity analysis restricted lung cancer cases to never-smokers.

**
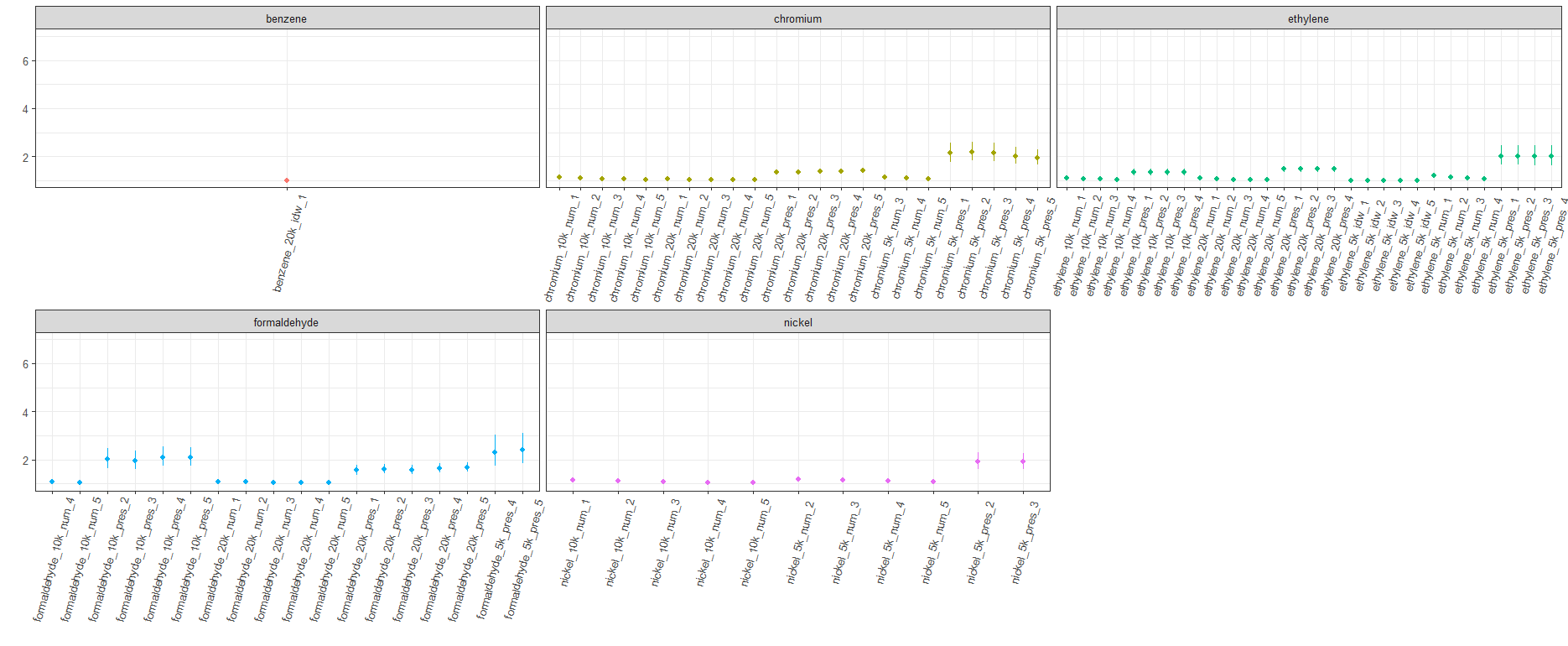
Figure S27.** Mean odds ratio and confidence interval for significant associations, breast cancer analysis.

**Note**: Mean odds ratio and confidence interval were formed calculating the harmonic mean over the three matching methods.

**
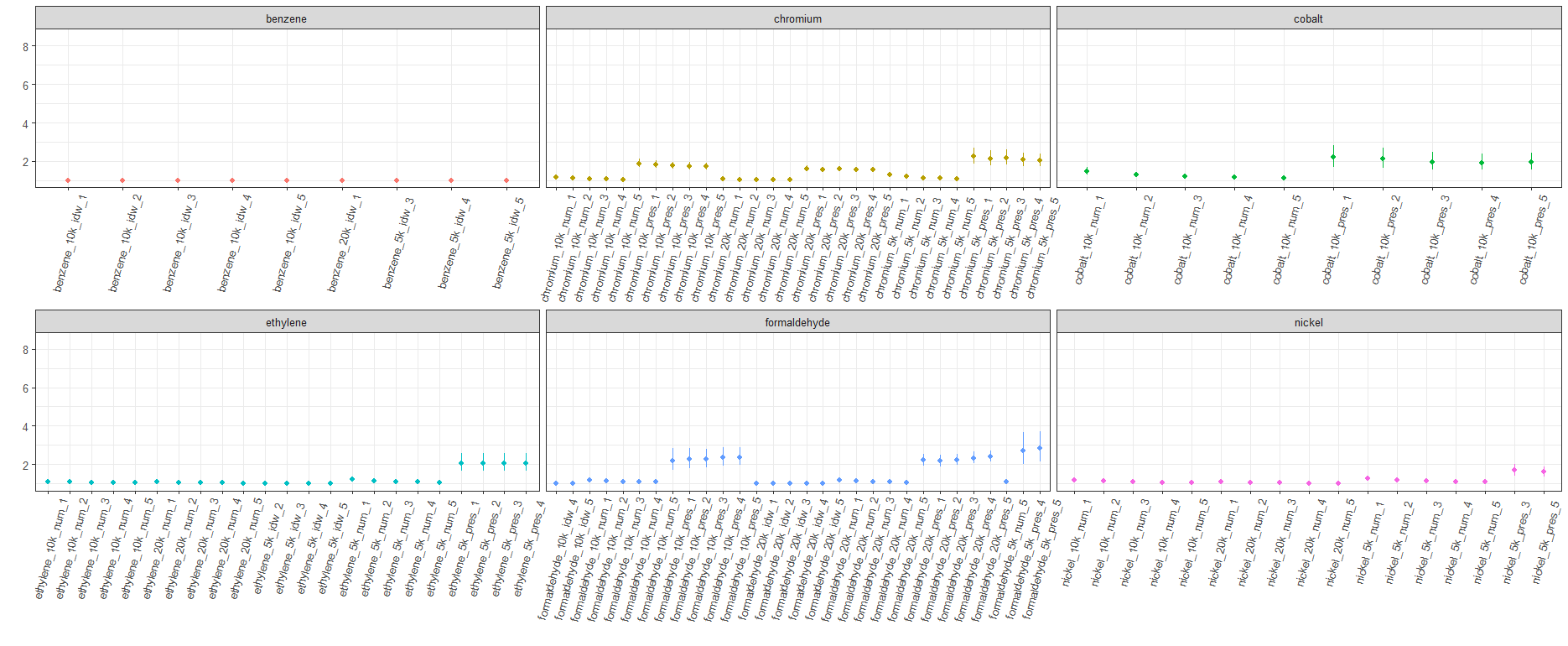
Figure S28.** Mean odds ratio and confidence interval for significant associations, lung cancer analysis.

**Note:** Mean odds ratio and confidence interval were formed calculating the harmonic mean over the three matching methods.


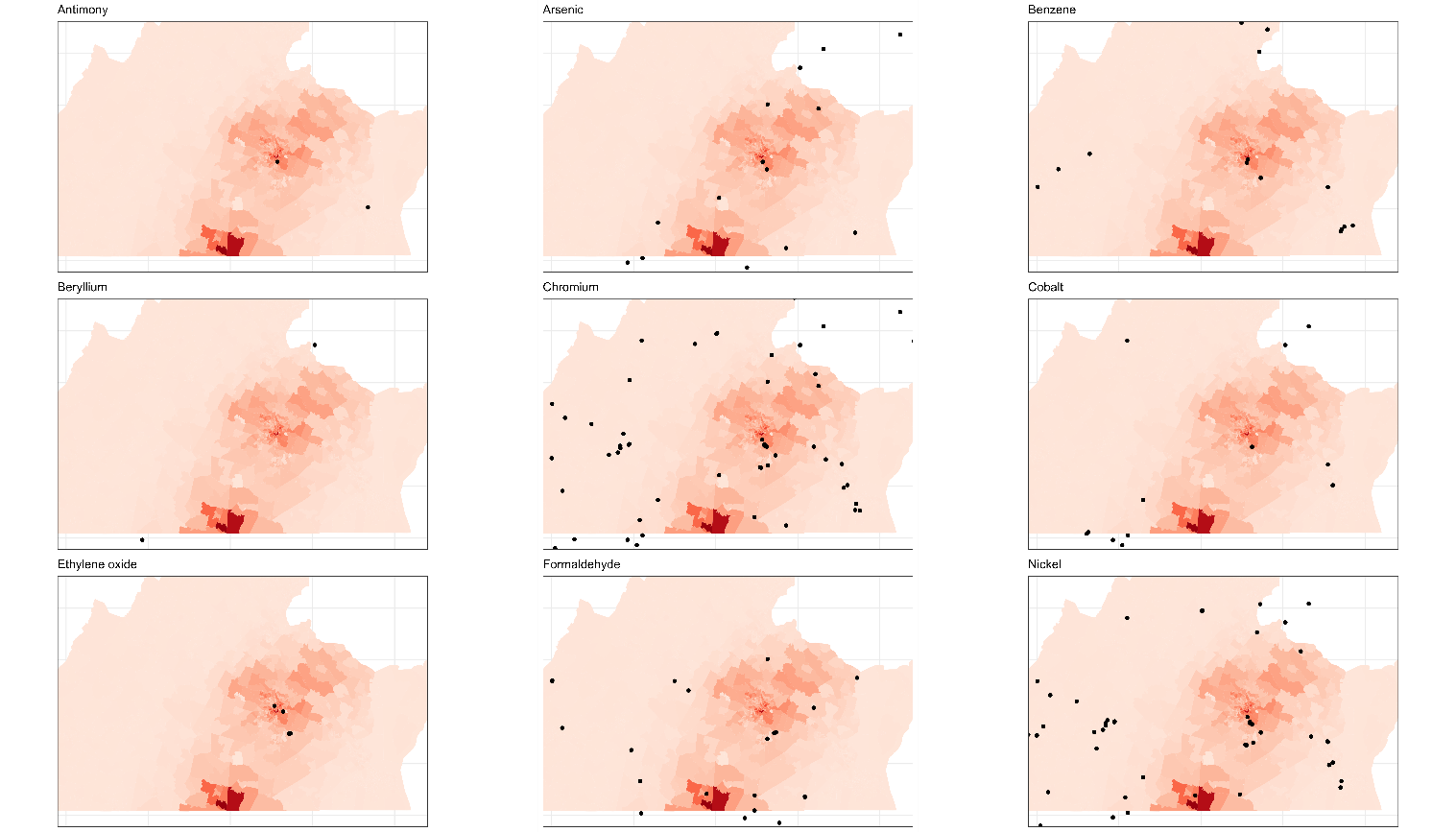
**Figure S29**. Geographic distribution of facilities emitting tested agents during study period.

**Note:** Maps zoomed in to emphasize catchment area for Massey Comprehensive Cancer Center. The number of participants in the overall sample (breast cancer cases, lung cancer cases and controls) was summed and aggregated to each census tract. Darker shades of red denote a greater number of study participants with residences in that census tract. Black circles denote emitting locations of facilities for each agent. Facilities emitting benzyl chloride, cadmium and cadmium compounds, and hydrazine were omitted from the plot due to very few facilities within the map extent.
